# Supplementary material for: Effects of Transcutaneous Electroacupuncture Stimulation (TEAS) on Eyeblink, EEG, and Heart Rate Variability (HRV): A Non-Parametric Statistical Study Investigating the Potential of TEAS to Modulate Physiological Markers
Source: Sensors (Basel). 2025 Jul 18;25(14):4468. doi: 10.3390/s25144468 (PMC12299896; doi:10.3390/s25144468)
Supplement: Supplementary file 1 [file sensors-25-04468-s001.zip › sensors-3670001-supplementary/sensors-3670001-supplementary.pdf]

Supplementary Material

Table S1. Subset of the 61 standard BLINKER indices used in this study, plus the ratio of left to right channels providing the best blinks (LRBR), as well as counts of left and right best blinks.

Base indices	Central tendency, dispersion
pAVRZ	mean, median, std, mad<br>goodMean, goodMedian<br>goodStd, goodMad
nAVRZ	mean, median, std, mad<br>goodMean, goodMedian<br>goodStd, goodMad
dZ	mean, median, std, mad<br>goodMean, goodMedian<br>goodStd, goodMad
dB	mean, median, std, mad<br>goodMean, goodMedian<br>goodStd, goodMad
dT	mean, median, std, mad<br>goodMean, goodMedian<br>goodStd, goodMad
dHZ	mean, median, std, mad<br>goodMean, goodMedian<br>goodStd, goodMad
dHB	mean, median, std, mad<br>goodMean, goodMedian<br>goodStd, goodMad
BpM	mean, goodMean
N_Blinks	Number of blinks
numberGoodBlinks	Number of good blinks
goodRatio	Ratio of good to all blinks
L1_R0	Blinks L (1) or R (0)
L_R_BlinkRatio	Ratio of left to right blink counts

Table S2. A. The 30 standard BLINKER indices for which, as time series, CEPS measures could be computed.

A. Standard BLINKER indices	Abbreviation
Velocity estimated by left tent line	AvLVA
Velocity estimated by right tent line	AvRVA
Tent closing time (Time between Intercept frames that define tent)	CTT
Zero closing time (Time between left Zero and max Frames)	CTZ
Blink duration (Left to Right Base)	dB
Half-Base duration: blink width at half the blink amplitude from left Base level	dHB
Half-Zero duration: blink width at half the blink amplitude from Zero level	dHZ

Tent duration, between intersections of down and upstroke linear fit lines with Zero line	dT
Zero duration, between right and left Zero	dZ
Correlation (R^2) between Upstroke and actual blink trajectory	leftR2
Slope of left tent line (NaN if no tent line)	leftSlope
Maximum value of blink peak	mV
Amplitude velocity ratio using interval from mV to Right Base	nAVRB
Amplitude velocity ratio using tent peak and slope of Right Tent line	nAVRT
Amplitude velocity ratio using interval from mV to Right Zero	nAVRZ
Maximum amplitude of Blink	pMB
Maximum height of Tent peak	pMT
Amplitude velocity ratio using interval from Left Base to mV	pAVRB
Amplitude velocity ratio using tent peak and slope of Left Tent line	pAVRT
Amplitude velocity ratio using interval from Left Zero to mV	pAVRZ
Reopening Time between X and Right X Intercept frames that define tent	reopTT
Time between Max and Right Zero frames	reopTZ
Correlation (R^2) between Downstroke and actual blink trajectory	rightR2
Slope of right tent line (NaN if no tent line)	rightSlope
Time that blink is at least 90% of its amplitude from left Base	tShutB
Time that blink is at least 90% of tent peak height	tShutT
Time that blink is at least 90% of its amplitude from left Zero	tShutZ
y-coordinate of intersection of left and right tent line with x-axis (NaN if no tent line)	y_int
Interval between successive Blink Max Amplitudes (peaks)	int_BMA
Interval between successive Blink Max positive Velocities (calculated from left Base)	int_BMVB

Table S2. B. The 34 CEPS measures derived for each of these BLINKER indices.

B. CEPS Family	Measure	Abbrev	Parameters used
Linear	Skewness		n/a
	Kurtosis		n/a
	root mean square of successive differences	RMSSD	n/a
	Hjorth Activity	HjorthA	n/a

	Hjorth Mobility	HjorthM	n/a
	Hjorth Complexity	HjorthC	n/a
	Absolute Jitter	jitta	n/a
	Local or relative Jitter	jitt	n/a
	Relative Average Perturbation (Jitter)	RAP	n/a
	5-Point Perturbation Quotient (Jitter)	PPQ5	n/a
Fractal	Petrosian Fractal dimension	P_FD	n/a
	Linden (Box) fractal dimension	LB_FD	n/a
	Sevcik fractal dimension	S_FD	n/a
Complexity	Allan factor *	AF	n/a
	Hurst Exponent	H	n/a
	Detrended Fluctuation Analysis Alpha	DFA α	n/a
	Asymmetry Index	ASI	n/a
	Complex Correlation Measure	CCM	2
	Standard Deviation along the minor axis of the Poincaré plot	SD1	n/a
	Standard Deviation along the major axis of the Poincaré plot	SD2	n/a
	Lempel-Ziv Complexity	LZC	n/a
	Permutation Jensen Shannon Complexity	PJSC	5, 2
Entropy	Tone entropy (entropy)		3
	Tone entropy (tone)		n/a
	Shannon Extropy	SEx	3, 2
	Multiscale Permutation Entropy	mPE	1, 1, 4
	Rényi Permutation Entropy	RPE	4, 2, 2
	Tsallis Permutation Entropy	TPE	4, 1, 1.1
	Edge Permutation Entropy	EPE	6, 1, 0.2
	Multiscale Permutation Min-Entropy	mPM_E	5, 1
	Composite Permutation Entropy Index	CPEI	1
	Distribution Entropy	DistEn	1, 8, 2
	Phase Entropy	PhEn	16

* Could not always be computed.

Further explanation of the BLINKER indices can be found in the original paper on BLINKER (Kleifges et al., 2017), and on the associated website, EEG-Blinks (http://vislab.github.io/EEG-Blinks/ [accessed 22 March 2024]. Further explanation of the CEPS measures can be found in the *Primer* included as part of the CEPS software package.

Demographics

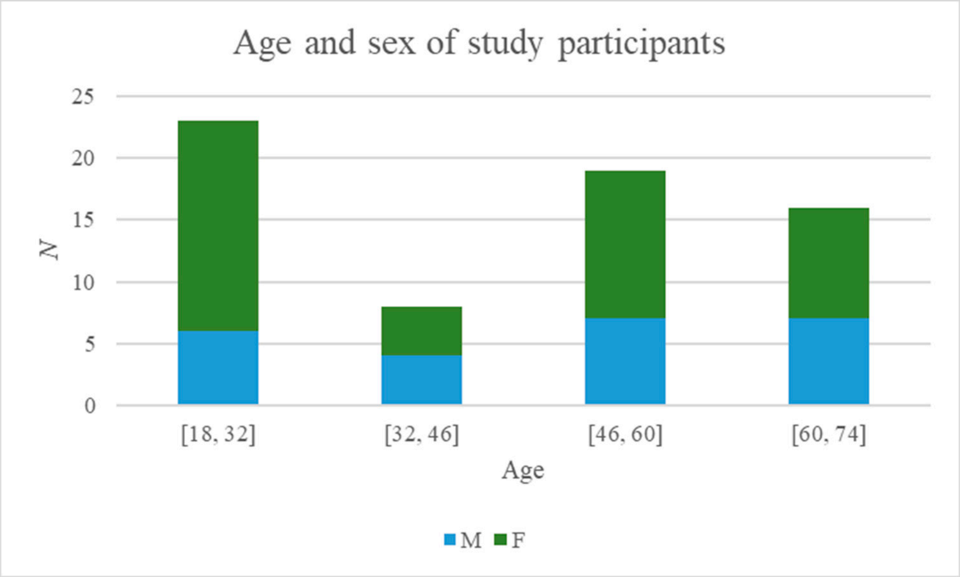

Figure S1. Sex and age distribution of study participants, from greenhorns to greys.

Top slicing

Table S3. 'Top 10' measures for BLINKER, using methods with and without repetition.

Top 10 'without repetitions'	Top 10 'with repetitions'	Overlap (6)
dHZ_mean	dHZ_mean	dHZ_mean
dHB_median	dHZ_mean	dHZ_median
dHZ_median	dHZ_mad	dHZ_mad
dHB_mean	dHB_median	dHB_mean
dHZ_mad	dHB_mean	dHB_median
dT_mean	dT_mean	dT_median
dT_median	dHZ_median	
dT_std	dHZ_median	
dT_mad	dHB_median	
dHB_mad	dT_median	

Table S4. Measures that occurred three times or more for *both* Values *and* Differences. (**Bold** = values or differences larger, red = max for that data type, highlighted in yellow = max for all data types. In square brackets, number with $W > 0.2$). The lower part of the Table shows measures that occurred only twice for both Values and Differences.

Data type	Measure	*N* values	Median *W* (values)	*N* diffs	Median *W* (diffs)
BLINKER	dHB_median	4	0.1048	4	0.1048
	dHZ_mad	3	0.1059	3	0.1059
	dHZ_mean	5	0.1275	4	**0.1285**
	dHZ_median	4	0.1085	3	**0.1136**
	dT_mean	4	0.1164	3	**0.1287**
Centroids	SpCen_O1	3	0.0558	3	0.0558
	SpCen_O2	3	**0.0672**	5	0.0623
	yPWR_C9	5	0.0619	3	**0.0679**
Cordance	LNzTAM_mT_Theta4_8_Cz	4	0.1072	3	**0.1111**
	SQzTAM_mT_Theta4_8_Cz	4	0.1100	3	**0.1144**

Hjorth parameters	HjorthA_Fz_1	5	0.0840	3	**0.1004**
	HjorthA_P3_1	4	0.0768	4	**0.0808**
	HjorthA_Pz_1	4	0.0995	3	**0.1111**
HRV	HFnu	6	0.0983	3	**0.1117**
	LFnu	5	0.1042	3	**0.1110**
	HF%	7	**0.1041**	5	0.1019
	LF%	4	0.0964	3	**0.1117**
	LF/HF	5	0.1046	3	**0.1118**
	SD2/SD1	6	0.0974	5	**0.0894**
	DFA α1	5	0.0753	4	**0.0968**
Mdn Pwr	O5_Cz	5	0.1635 [1]	3	**0.2120** [2]
Regional Ratios	Alph_F1_sham_P_A_a	4	0.0596	3	**0.0637**
	Alph_F1_sham_P_A_r	4	0.0557	4	**0.0649**
Asymm L	P_C_5_ASP_r	5	**0.1396**	4	0.0987 [1]
Asymm Ratio	Periph_Cent_5	4	**0.1035**	4	0.0987 [1]
	Periph_Cent_O5_r	3	**0.1196**	3	0.0953 [1]
Temperature	TempSlope	19	**0.1236** [4]*	7	0.01767
	TempSlope V	9	**0.2040** [5]	13	0.06409
			[3 red]		[7 red]
**Not eligible**	(as only in top 10 for 2 comparisons)		**Max *W***		**Max *W***
BLINKER-CEPS	PJSC_cTZ [Slot by Sess: 5]	2	0.1539		
	jitt_leftR2 [Sess by Slot 1]			2	0.1377
1 Hz bins	P8_7.5_I_PS_mT [Se by Sl 1]	2	0.3182	2	**0.3367**
	P3_2.5_A_PS_mT [St by Sl 2.5]	2	**0.3173**	2	0.3155
Asymm 171	P3_O2_O2.5_LnASP_r [St by Sl 2.5]	2	0.1798		
	P4_O1_O2.5_LnASP_r [Sl by St 5]			2	0.2006
Wackermann	Phi_5_A_TR [Slot by Sess: 6]	2	0.1453		
	mdn_Sigma_all_A_TR [St by Sl 10]			2	0.0228

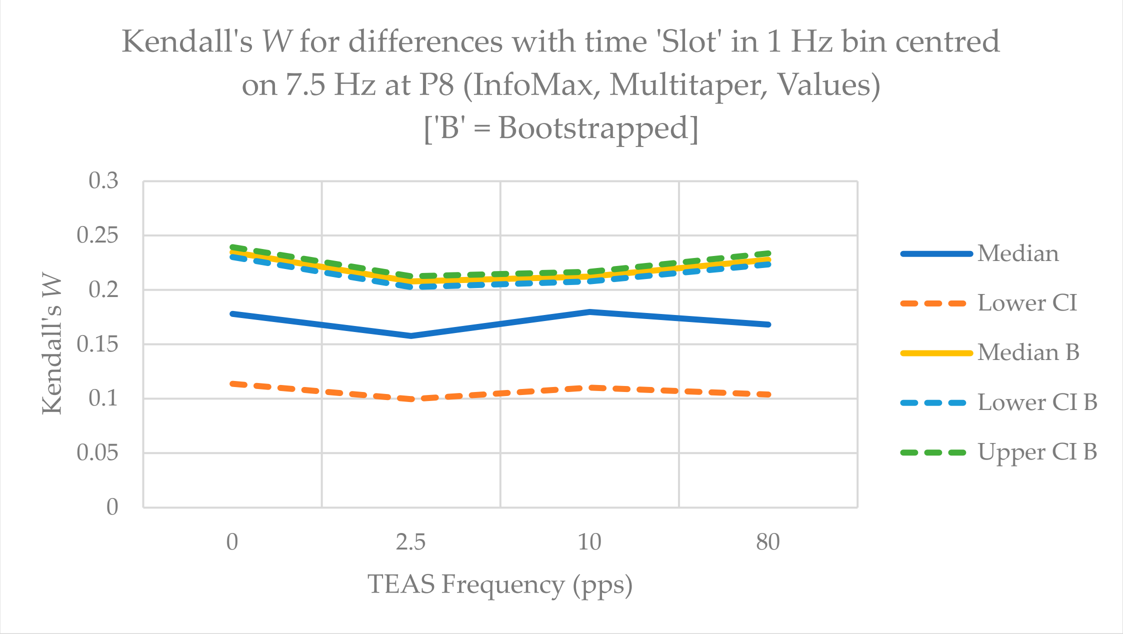

Figure S2. Median values and 95% confidence intervals of Kendall's *W* for differences between time 'Slots' at each stimulation frequency, for the EEG 1 Hz 'bin' centred on 7.5 Hz at channel P8 (one of the measures listed in Table S4). For the non-bootstrapped results, the upper confidence interval = 1, so is not shown.

Table S5. The ten CEPS measures and paired BLINKER data types that provided greatest differentiation between time Slots using Conover-Iman tests, with corresponding results for ten of the BLINKER measures themselves in the final two columns. Largest 95th percentiles of the Conover-Iman statistic (CIS) are shown, in decreasing order in each column.

CEPS measures	95%	Data type	95%	BLINKER	95%
Jitta	3.539	dT	2.552	dHZ	3.786
RMSSD	3.481	mV	2.508	dT	3.696
Hjorth A	3.384	pAVRT	2.476	dHB	3.666
SD1	3.191	pMB	2.454	dZ	3.178
SD2	3.144	reopTT	2.434	goodRatio	3.015
Jitt	3.126	leftR2	2.413	pAVRZ	2.741
Jitter RAP	3.098	dHB	2.385	N_Blinks	2.514
Tone-Entropy E	2.995	dHZ	2.37	nAVRZ	2.354
Jitter PPQ5	2.917	y_int	2.367	dB	2.080
Tone-Entropy T	2.892	pMT	2.363	BpM	2.064

Table S6. The ten CEPS measures and paired BLINKER data types that provided greatest differentiation between stimulation Frequencies using Conover-Iman tests, with corresponding results for ten of the BLINKER measures themselves in the final two columns. Largest 95th percentiles of the Conover-Iman statistic (CIS) are shown.

CEPS measures	95%	Data type	95%	BLINKER	95%
LZC	2.191	pAVRB	2.190	dT	2.213
ASI	2.144	pAVRZ	2.188	dZ	2.211
Skewness	2.126	y_int	2.060	dHZ	2.188
H	2.125	pMT	2.028	dB	2.156
RPE	2.104	pAVRT	2.024	BpM	2.096
TPE	2.062	tShutT	2.013	dHB	2.047
PhEn	2.061	cTT	2.010	nAVRZ	2.031
SD2	2.060	rightSlope	2.006	pAVRZ	1.984
Kurtosis	2.029	pMB	1.999	goodRatio	1.851
Jitter RAP	2.024	R_AvRVA	1.993	N_Blinks	1.846

Table S7. The ten CEPS measures and paired BLINKER data types that provided greatest differentiation between Sessions using Conover-Iman tests, with corresponding results for ten of the BLINKER measures themselves in the final two columns. Largest 95th percentiles of the Conover-Iman statistic (CIS) are shown.

CEPS measures	95%	Data type	95%	BLINKER	95%
PJSC	2.640	leftSlope	2.169	nAVRZ	2.255
Shimmer	2.268	dZ	2.162	pAVRZ	2.136
CPEI	2.245	R_AvLVA	2.157	BpM	2.112

mPE	2.201	mV	2.105	N_Blinks	2.018
Shimmer_apq5	2.179	tShutB	2.094	dB	1.942
FD_Linden_Box	2.158	pMB	2.092	dZ	1.856
AF	2.155	leftR2	2.077	goodRatio	1.833
Shimmer_apq3	2.148	tShutZ	2.076	dT	1.748
LZC	2.146	tShutT	2.068	dHB	1.733
Petrosian_FD	2.134	y_int	2.062	dHZ	1.634

In **Tables S5-S7,** the largest 95th percentiles of the Conover-Iman statistic (CIS) are more commonly greater for the CEPS measures than for the BLINKER measures, as illustrated in **Figure S3**.

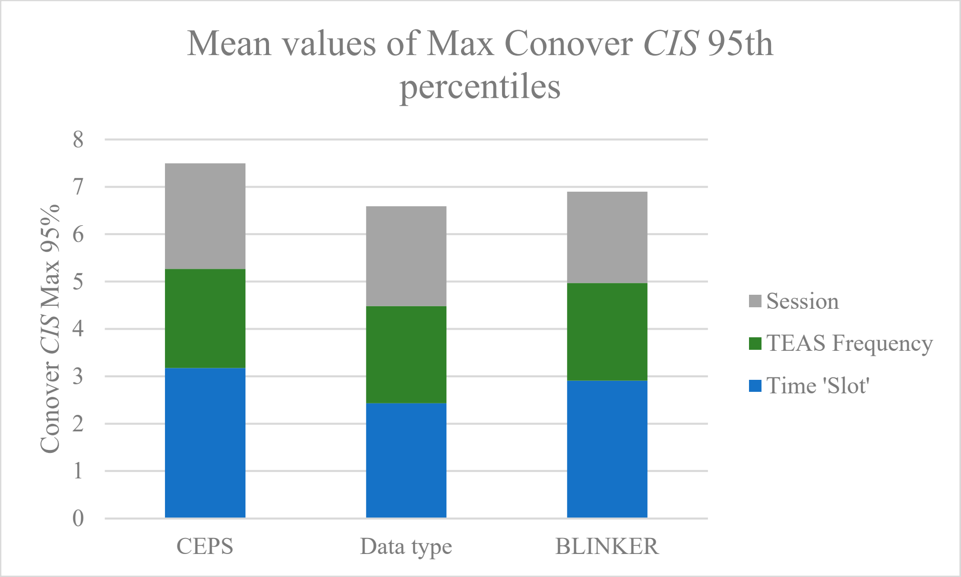

Figure S3. Visual summary of **Tables S5-S7,** comparing mean values of the Maximum 95th percentiles of the Conover-Iman statistic (CIS) for the top 10 measures that provided greatest differentiation between time Slots, Frequencies and Sessions.

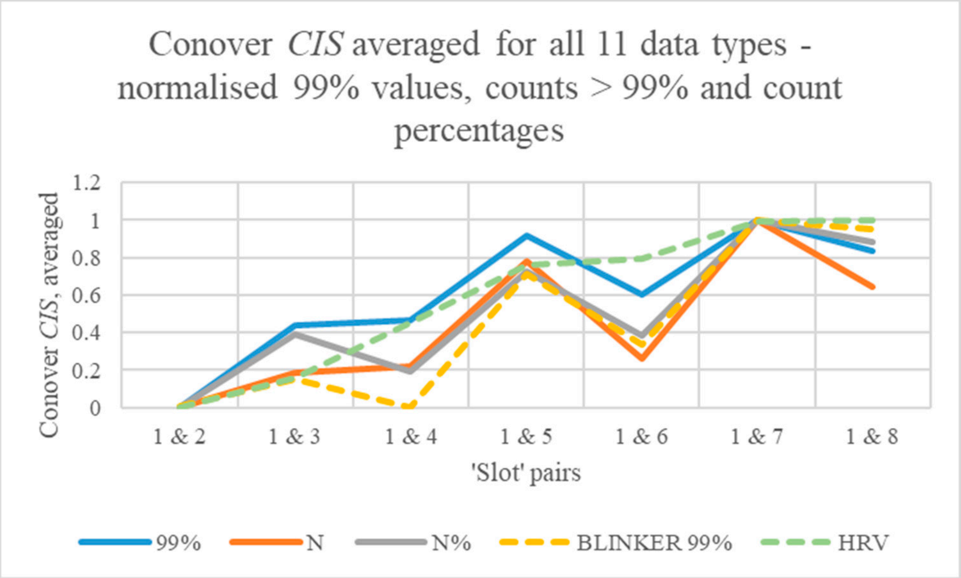

Figure S4. Min-max normalised results for the Conover-Iman statistic (CIS) (99th percentile values, counts of values > 99%, and percentages of these counts of all the differences analysed), averaged for 11 data types analysed, and comparing differences over time within sessions.

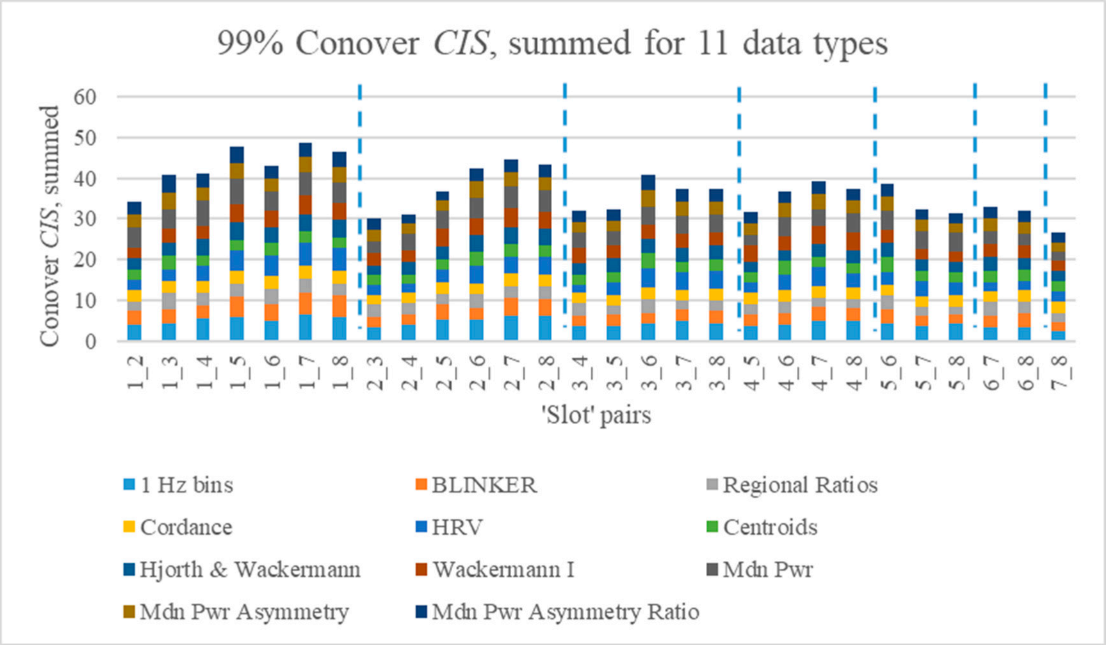

Figure S5. Conover *S* for all 28 Slot pairs, with 99% values stacked rather than averaged, emphasising the effects of time and of combining results for all the 11 data types.

Note the consistent increases from Slots 1, 2, 3 and 4 (before and during stimulation), and the subsequent decreases from Slots 5, 6 and 7 (post-stimulation).

Table S8. Measures and indices most affected by stimulation Frequency, time Slot and Session. Frequency, time Slot or Session pairs that are the same for both measures for a given data type are shown in bold type.

Data type		Max CIS	Pair	Next to max CIS	Pair
1 Hz bins	Slot	P3_2.5_A_PS_mT	4-6	P3_2.5_A_PS_mT	4-7
	Session	O1_9.0_I_PS_mT	**1-4**	O2_10.0_I_PS_mT	**1-4**
	pps	Cz_5.0_I_PS_mT	2.5-80	C3_30.0_A_PS_mT	0-10
BLINKER	Slot	dHZ_median	1-7	dT_median	1-8
	Session	dB_mean	1-2	N_Blinks	1-4
	pps	dB_goodMedian	0-80	dT_goodMean	2.5-80
Regional Ratios	Slot	Theta_C_O_r	1-6	I2_20_P_A_a	1-3
	Session	Theta_sham_P_A_a	1-3	I5_18.._sham_C_O_a	1-2
	pps	Theta_F1_sham_P_A_r	2.5-80	Alph_F1_sham_C_O_r	0-10
Cordance	Slot	LNzTAM_mT_Theta_P8	**2-7**	SQzTAM_mT_Theta_P8	**2-7**
	Session	LNNoInf_CMW_Delta_Fz	**1-4**	LNNoAM_mT_Alpha_C4	**1-4**
	pps	SQNoAM_mT_Theta_F3	**0-80**	LNNoAM_mT_Theta_F3	**0-80**

Kubios	Slot	SD2/SD1	3-7	SD2/SD1	2-7
	Session	D6_trend_pc_ECG1	1-3	D3_trend_pc_BVP1	2-4
	pps	SD2/SD1	0-80	SD2/SD1	10-80
Centroids	Slot	SpCen_Fp2	5-6	yPWR_C9	3-6
	Session	SpCen_P8_0	2-4	SpCen_P4_0	1-3
	pps	xFREQ_A7	0-10	xPWR_L8	2.5-80
Hjorth & Wack'n	Slot	HjorthA_Fz	1-7	HjorthA_Cz	1-5
	Session	HjorthM_P7	1-4	HjorthM_P8	2-4
	pps	HjorthC_C4	0-2.5	HjorthA_Cz	2.5-10
Wack I all	Slot	mdn_Sigma_all_A_TR	2-8	mdn_Sigma_all_A_TR	1-7 / 8
	Session	Sigma_75_A_PS	**1-3**	Phi_5_I_PS	**1-3**
	pps	Phi_55_I_TR	0-2.5	Sigma_53_A_PS	2.5-80
Mdn Pr	Slot	O2.5_P3	4-8	O5_P3	1-5
	Session	O10_O1	**1-4**	Pz_alpha	**1-4**
	pps	O5_Cz	2.5-80	O30_C4	10-80
Mdn Pr A	Slot	Cz_O1_O2.5_LnASP	**4-8**	Cz_O1_O2.5_LnASP_r	**4-8**
	Session	P4_P3_O30	1-2	Post_Ant_O5	1-3
	pps	P_C_5_ASP_r	2.5-80	P_C_7.5_ASP_r	2.5-10
Mdn Pr AR	Slot	Periph_Cent_O5_r	2-8	Periph_Cent_5	2-7
	Session	Post_Ant_theta_r	1-3	P4_P3_Beta3_r	1-2
	pps	Periph_Cent_O7.5_r	0-2.5	Periph_Cent_5	2.5-80

Differences over time (between Slots) within Sessions

For the majority of data types considered (8 out of 11), the 99th percentile of Kendall's *W* is greatest for changes over time (i.e., when comparing time Slots *within* sessions than when comparing results for the four different Sessions or the four Stimulation frequencies, as can be seen in **Figure S6**).

From **Figure S6** it is clear that Kendall's *W* as a measure of effect size was most commonly very small (< 0.1), although > 0.1 for five data types (including BLINKER) for Time Slot comparisons, and for the Frequency and Session comparisons for Cordance only.

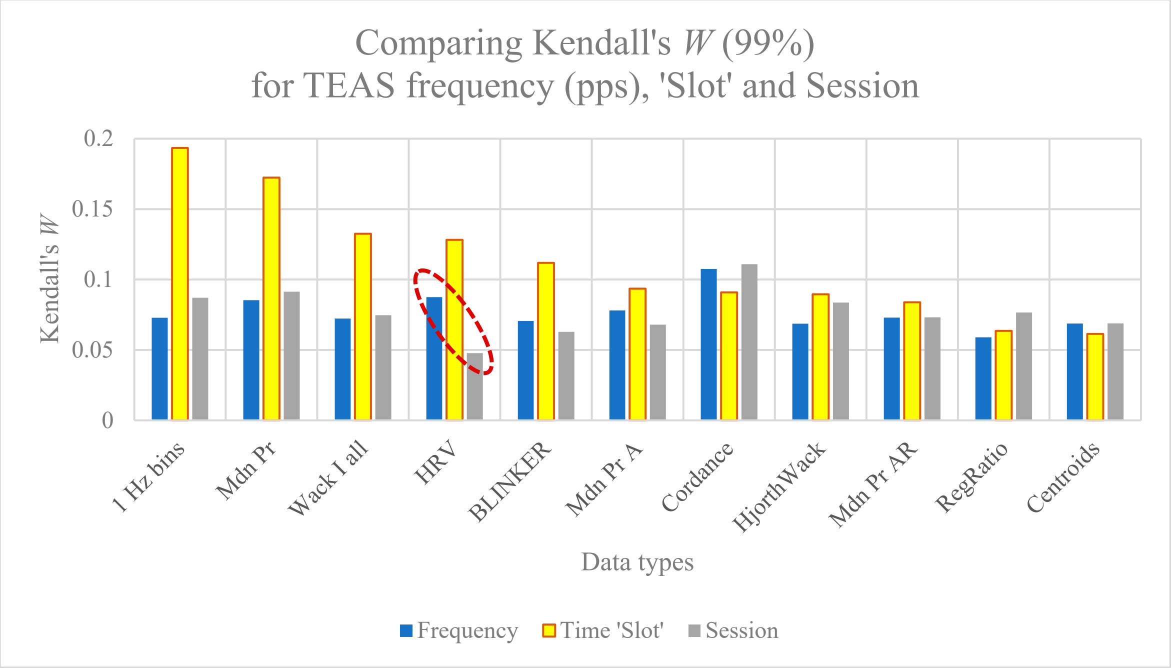

Figure S6. Comparing Effect sizes (Kendall's *W*) for TEAS frequency, 'Slot' and Session. Note that for the majority of data types considered (8 out of 11), the 99th percentile of Kendall's *W* is greatest for changes over time (i.e., when comparing time Slots *within* sessions than when comparing results for the four different Sessions or the four Stimulation frequencies.

Appendix S1 – Some Conover-Iman (CIS) results, focusing on the EEG median power in 1 Hz bins

Values and counts of the 99.9th percentiles of the Conover-Iman statistic when comparing eyeblink, HRV and nine other EEG-derived measures for the four stimulation frequencies, eight time Slots and four Sessions are shown in the **Table S9** (compare the graphical summary in **Figure S7**).

Table S9. Values and counts of the 99.9th percentiles of the Conover-Iman statistic (CIS) when comparing eyeblink, HRV and the nine other EEG-derived measures for the four stimulation frequencies, eight time Slots and four Sessions. Also included, counts of CIS > 5, etc. Top four values for Slots, Sessions and Frequencies (pps) are shown in bold (red for Slots, Black for Sessions and Green for Frequencies).

Data type	Analysis	*N* rows	99.9%	N>99.9%	N > 5	N > 4	N > 3	N > 2
1 Hz bins	Slot	8512	**6.686**	239	2782	7954	20562	50935
	Session	17024	**3.967**	103	0	98	1211	8468
	pps	17024	**3.893**	91	12	74	543	5426
BLINKER	Slot	268	**5.254**	7	12	103	405	1377
	Session	536	3.256	3	0	0	9	138
	pps	536	3.534	3	0	0	14	185
RegRatio	Slot	252	4.051	8	0	9	110	605
	Session	504	**3.678**	4	0	1	26	162
	pps	504	3.255	4	0	0	11	144
Cordance	Slot	11184	3.545	314	0	72	1601	17969
	Session	22368	3.404	135	0	17	473	6432
	pps	22368	3.396	132	0	20	505	6578

Kubios HRV	Slot	144	**5.866**	5	34	103	305	813
	Session	2416	3.233	15	0	0	35	633
	pps	288	**3.929**	2	1	2	24	163
Centroids	Slot	188	3.831	6	0	4	51	440
	Session	376	3.351	3	0	2	11	132
	pps	376	3.522	3	0	0	14	116
Hjorth Wackermann	Slot	276	4.817	8	4	33	236	1059
	Session	552	**4.194**	4	0	5	36	255
	pps	552	3.443	4	0	1	16	193
Wackermann I	Slot	3188	**5.124**	90	113	876	3974	13732
	Session	6376	3.200	39	0	3	94	1557
	pps	6376	3.276	39	0	1	98	1711
Mdn Pwr	Slot	2280	**6.754**	64	502	1560	4327	11926
	Session	4560	**4.112**	28	3	38	370	2292
	pps	4560	**4.209**	28	5	45	255	1774
Mdn Pwr Asymmetry	Slot	2736	4.358	17	4	48	374	1846
	Session	4656	3.532	28	0	6	122	1406
	pps	4656	**4.050**	28	0	32	207	1640
Mdn Pwr Asymmetry Ratio	Slot	2120	4.773	60	40	172	747	4172
	Session	4240	3.530	26	0	3	108	1242
	pps	4240	3.842	26	1	22	151	1410

Note that 1 Hz bins and Median power both resulted in top four 99.9th percentile values of CIS for all three comparisons (Slot, Session and Frequency), Kubios HRV for two, and BLINKER, Regional ratios, the Hjorth and Wackermann measures and Median Power Asymmetry only for one. Cordance, the Centroids and the Median Power Asymmetry ratios did not result in any (no coloured text in the '99.9%' column).

Figure S7 illustrates the maximum 95th percentiles of the Conover-Iman statistic when comparing eyeblink, HRV and the nine other EEG-derived measures for the four stimulation frequencies, eight time Slots and four Sessions.

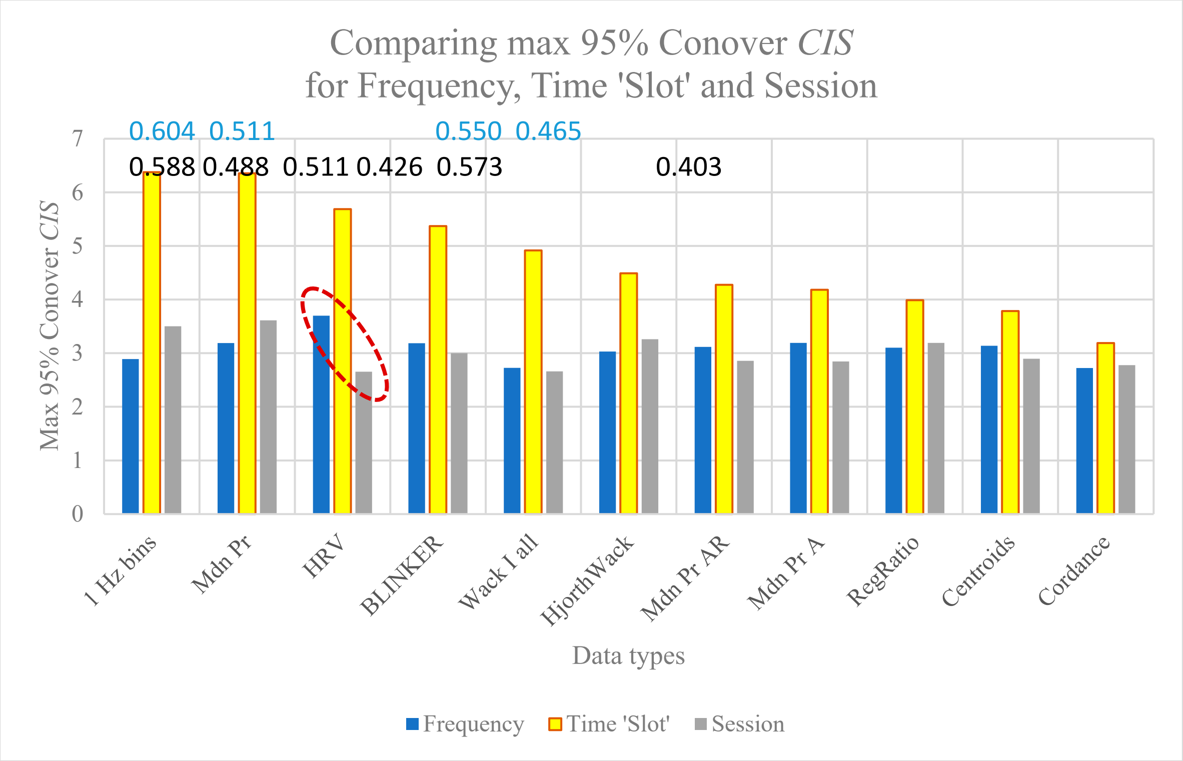

Figure S7. Maximum 95th percentiles of the CIS – rather than 99.9th percentiles – when comparing eyeblink, HRV and the nine other EEG-derived measures for the four stimulation frequencies, eight time Slots and four Sessions. Largest percentiles for Slot are on the left, smallest on the right. Mann-Whitney test effect sizes (ES) > 0.4 are shown in blue type for comparisons between frequencies and Slots, and in black type for comparisons between Slots and Sessions. Greatest differences in maximum 95% CIS between frequencies and sessions occurred for Kubios HRV(although with p > 0.05), as indicated by the red oval.

Focusing on the 1 Hz bins results as an example:

1 Hz bins spaced 2.5 Hz apart provide the best results, as might be expected, given that stimulation frequencies were all multiples of 2.5 pps. while 1 Hz bins spaced 15 Hz apart the worst results, in terms of differentiating stimulation frequency. Unexpectedly, however, 1 Hz bins spaced 30 Hz apart performed well (**Figure S8**). The reason for this is unknown.

Figure S9 illustrates that, of the EEG channels, Cz was the most sensitive to stimulation frequency effects, Fp1 the worst (bearing in mind that BLINKER often used Fp1 or Fp2 for blink parameters). Apart from the frontal and occipital electrodes (which both, like T7, P7 and Pz, gave poor results), results were better on the right than on the left.

Differences over time are illustrated in **Figure S10**. There was already a difference between the stimulation frequency groups at baseline, as observed with much of the other analysis undertaken, but greatest difference was during Slot 2. Post-stimulation, differences decreased markedly, but with a possible ‘rebound’ effect in Slot 8.

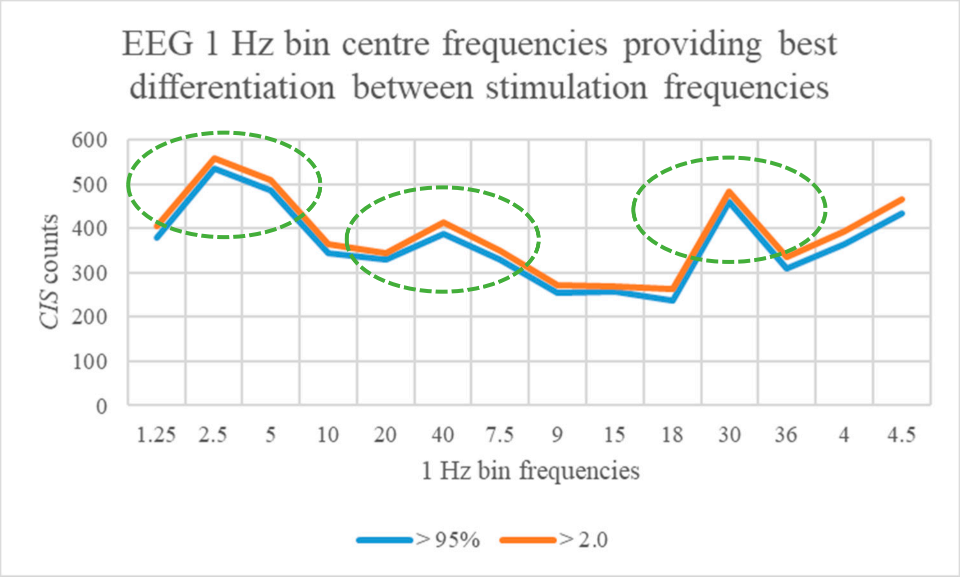

Figure S8. Counts of 95th percentile Conover *S* for EEG 1 Hz bin centre frequencies providing best differentiation between stimulation frequencies.

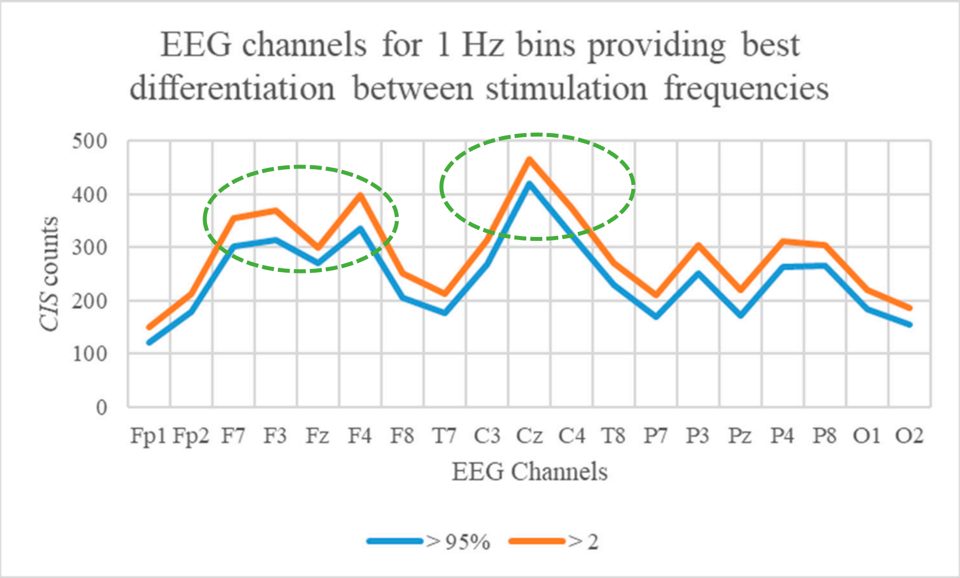

Figure S9. Counts of 95th percentile Conover *CIS* for EEG channels for 1 Hz bins providing best differentiation between stimulation frequencies.

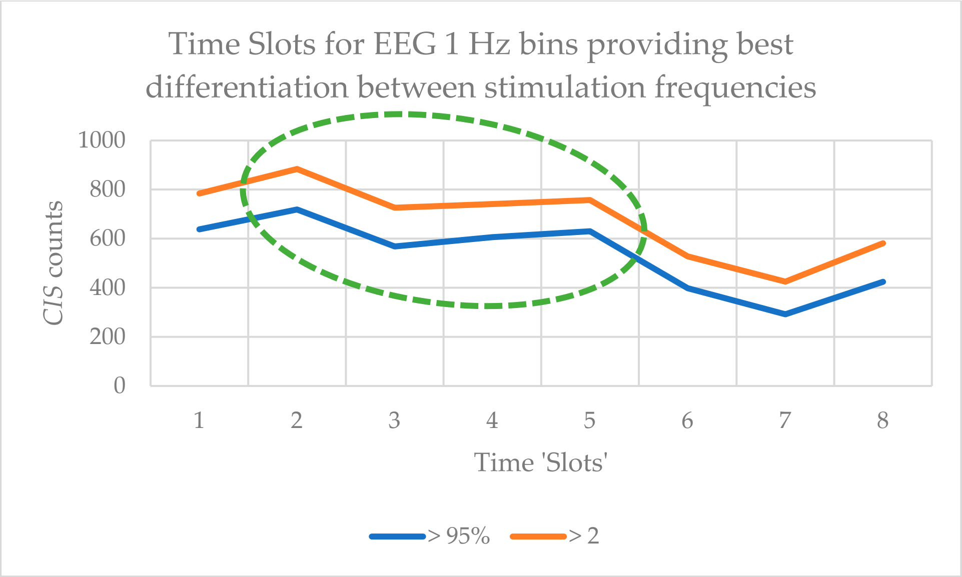

Figure S10. Counts of 95th percentile Conover *S* for time Slots for EEG 1 Hz bins providing best differentiation between stimulation frequencies.

The 1 Hz bins were created using two different pre-processing methods, as mentioned above (EEGLab/MARA and a novel method based on machine learning developed by Thea Radüntz). For differentiation between stimulation frequencies, the former resulted in 85 counts of CIS > 99.9%, but the latter only four. Clearly the more established method was more useful in this context.

Most of our EEG data types were based on Extended Infomax ICA, but we also used AMICA for the 1 Hz bins. However, there were only 39 occurrences of Infomax results with CIS > 99.9%, as opposed to 63 for AMICA. As Scott Makeig has written [EEGLab List email 05.02.23]: AMICA "is still to our knowledge (and testing) the most effective ICA method applied to EEG data". In a further comparison, using Thomson Multitaper [63] for time-frequency decomposition differentiated better between stimulation frequencies than continuous Morlet wavelet [64].

Appendix S2. Graphical comparisons of results using measure Values and 'Differences' (Values normalised with respect to baseline, within each Session)

II.1. BLINKER and CEPS-BLINKER

For all measures considered together, median Friedman's Chi^2 for comparisons between the Stimulation frequencies was greater for Differences than for Values, in all Slots (**Figure S11**).

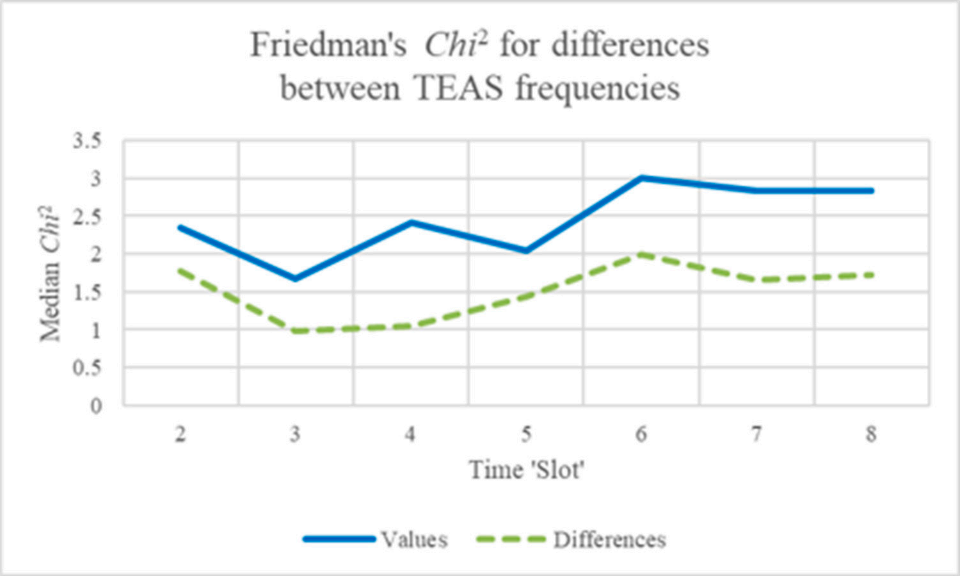

Figure S11. Median Friedman's *Chi*^{2} for comparisons between the Stimulation frequencies, for all BLINKER and CEPS-BLINKER measures, using non-normalised and normalised measure Values ('Differences') in the different time Slots.

In contrast, effect size (Kendall's *W*) was sometimes greater for Values, and sometimes greater for Differences (**Figure S12**).

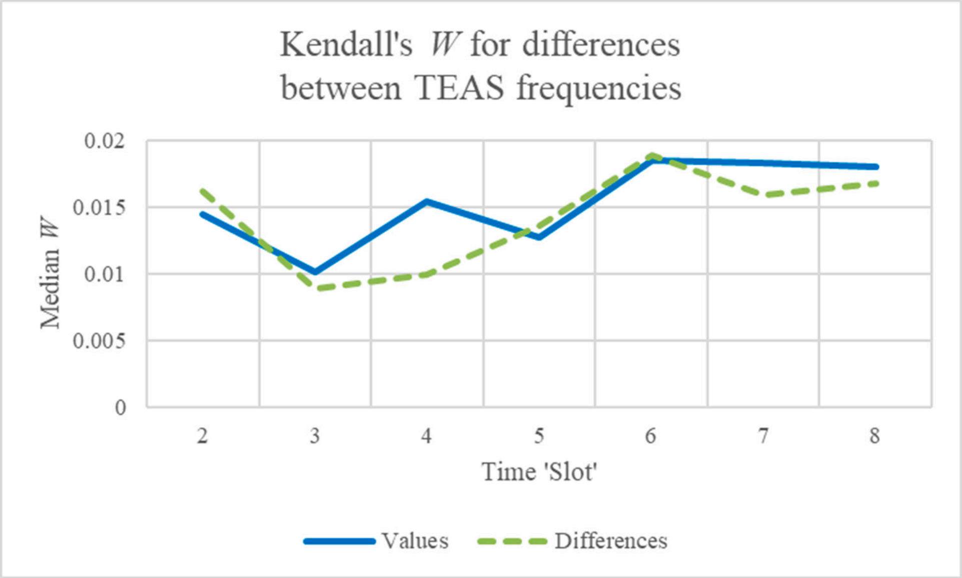

Figure S12. Median effect size (Kendall's *W*) for comparisons between the Stimulation frequencies, for all BLINKER and CEPS-BLINKER measures, using non-normalised and normalised measure Values ('Differences').

However, as a counter-example, comparisons between time Slots for the different Stimulation frequencies showed greater median values of Chi-square for Values than for Differences, with a similar finding for Kendall's *W* (**Figures S13**, **S14**).

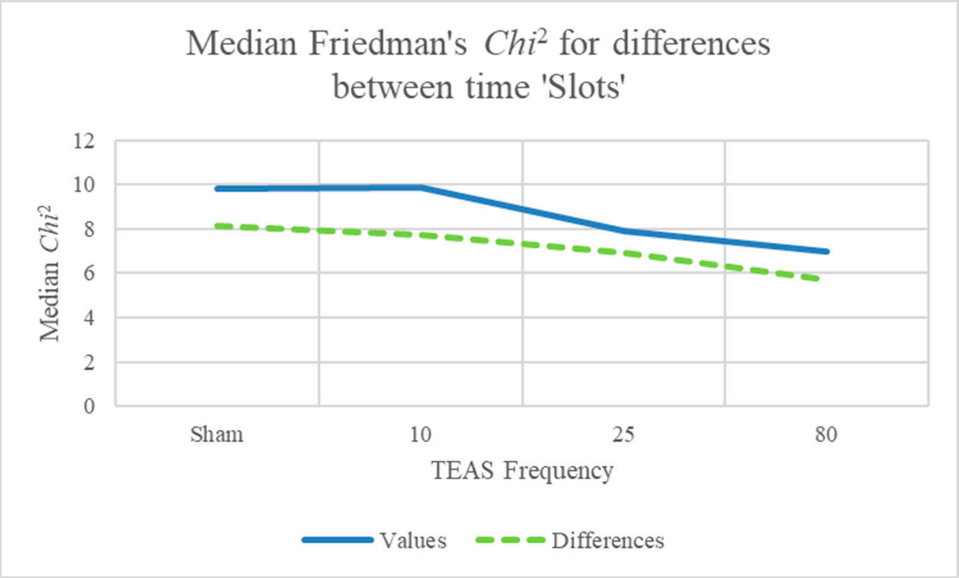

Figure S13. Median Friedman's Chi-square for comparisons between time Slots, for all BLINKER and CEPS-BLINKER measures at the different stimulation frequencies, using non-normalised and normalised measure Values ('Differences').

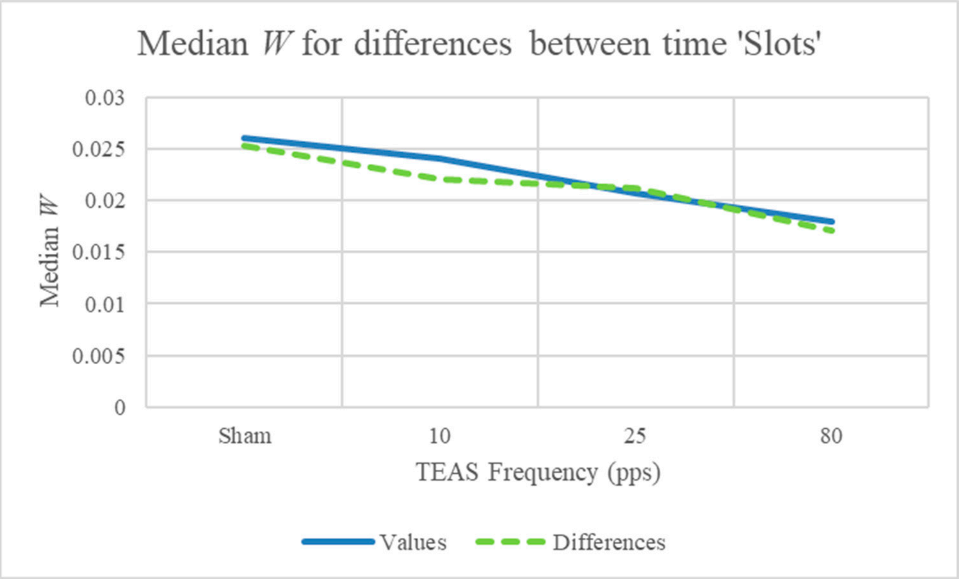

Figure S14. Median effect size (Kendall's W) for comparisons between time Slots, for all BLINKER and CEPS-BLINKER measures at the different stimulation frequencies, using non-normalised and normalised measure Values ('Differences').

Note that for both Sham and 10 pps stimulation, differences over time (Chi-square and W) were greater for the Slot values than for the Slot differences.

For comparisons between Sessions in the different time Slots, again median values of Chi-square are greater for Values than for Differences, with a similar finding for Kendall's W – except in time Slot 6 (**Figures S15**, **S16**).

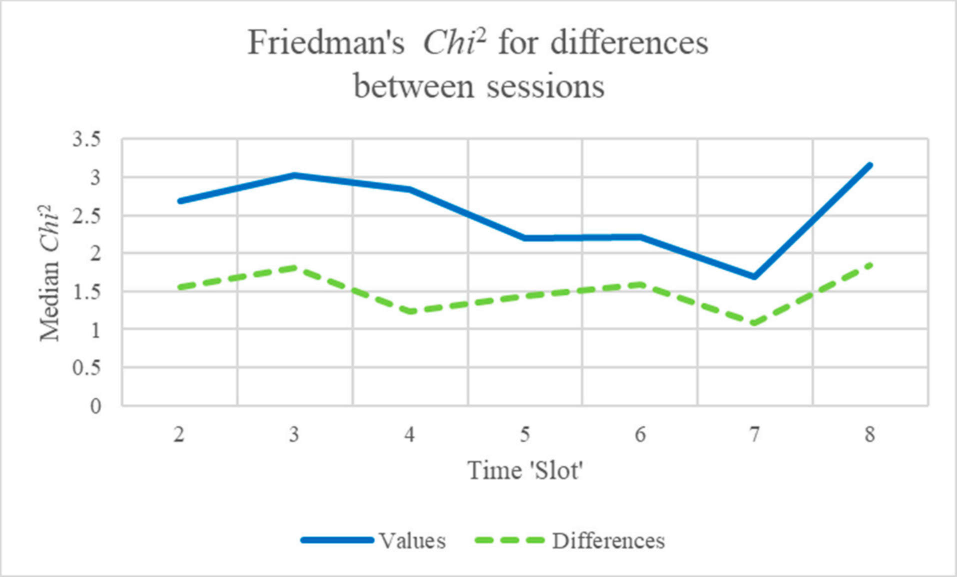

Figure S15. Median Friedman's Chi-square for comparisons between Sessions, for all BLINKER and CEPS-BLINKER measures in the different time Slots using non-normalised and normalised measure Values ('Differences').

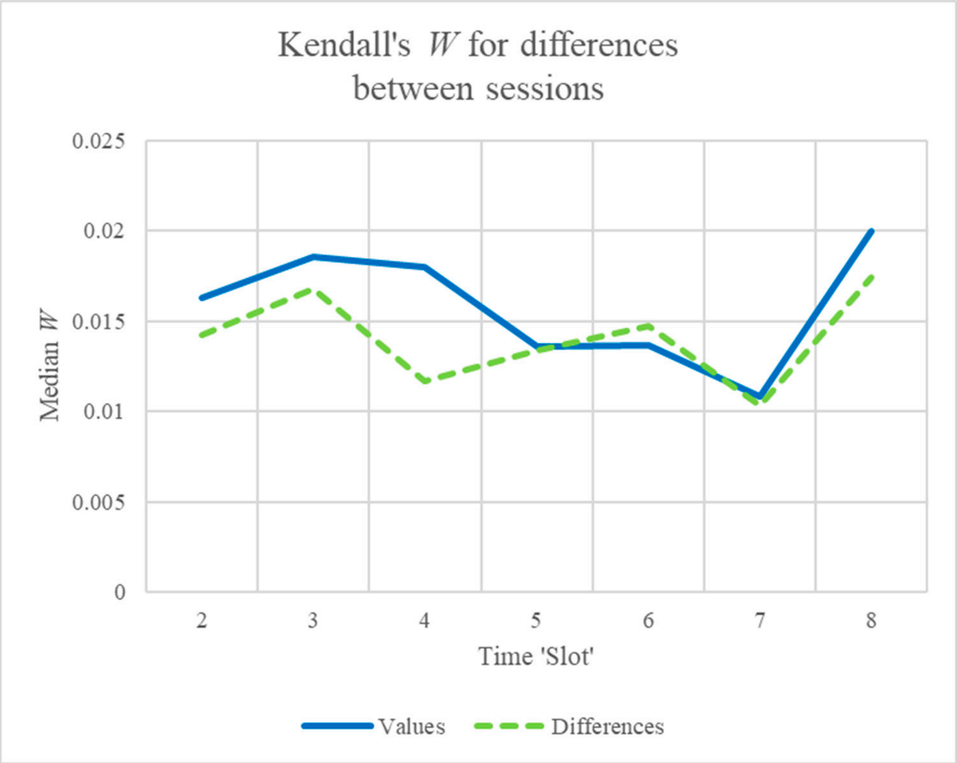

Figure S16. Median effect size (Kendall's *W*) for comparisons between Sessions, for all BLINKER and CEPS-BLINKER measures in the different time Slots using non-normalised and normalised measure Values ('Differences').

Corresponding findings for the HRV measures are shown in the following Figures: **S17** and **S18** for comparisons between the Stimulation frequencies in Slots, **S19** and **S20** for comparisons between time Slots for the different Stimulation frequencies, **S21** and **S22** for comparisons between Sessions in

the different time Slots. HRV measures not output using both the Differences and Values methods were excluded (e.g. Recurrence plot measures and MSE scales for Differences, and EDR for Values).

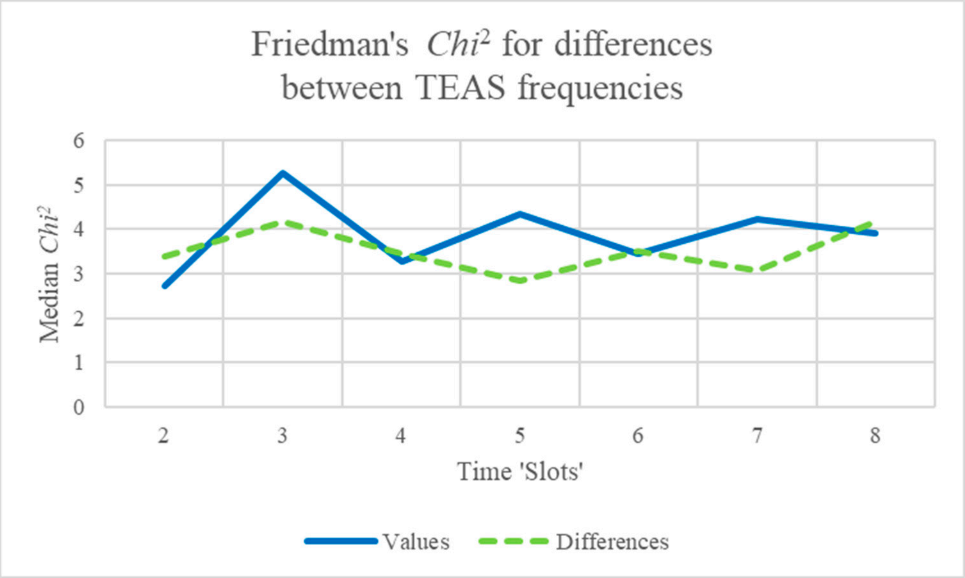

Figure S17. Median Friedman's Chi-square for comparisons between time Stimulation frequencies, for the HRV measures in the different time Slots, using non-normalised and normalised measure Values ('Differences').

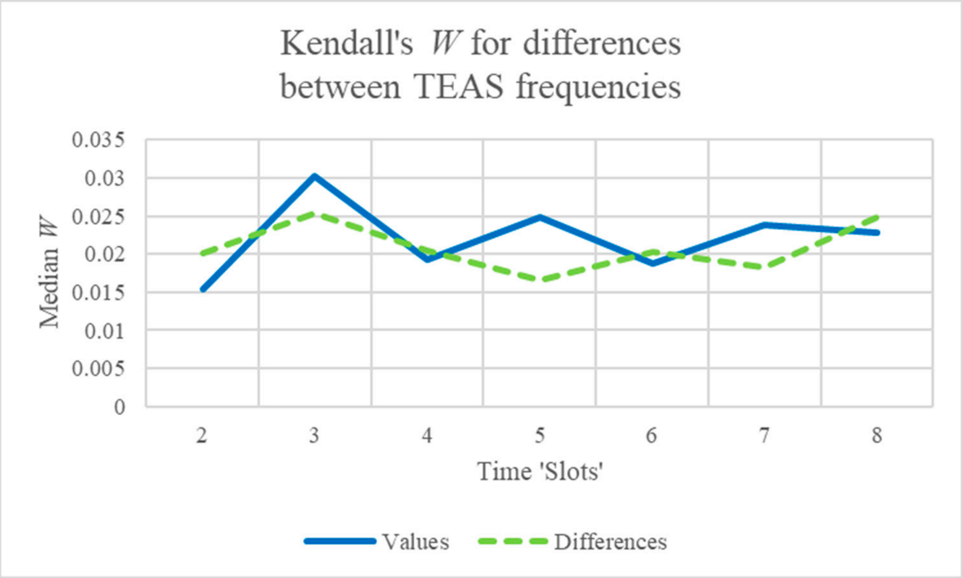

Figure S18. Median effect size (Kendall's *W*) for comparisons between time Stimulation frequencies, for the HRV measures in the different time Slots, using non-normalised and normalised measure Values ('Differences').

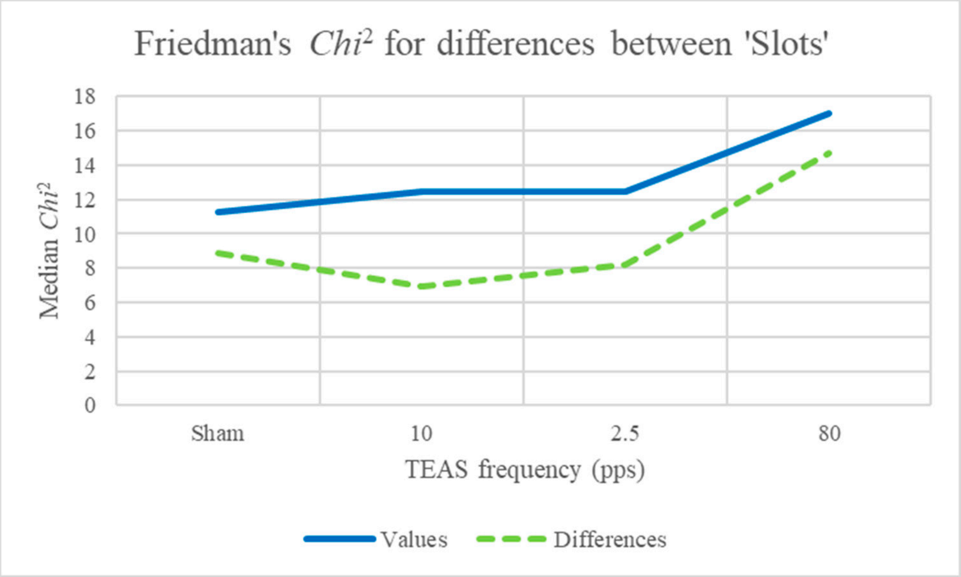

Figure S19. Median values of Friedman's Chi-square for comparisons between time Slots, for HRV measures at the different stimulation frequencies, using non-normalised and normalised measure Values ('Differences').

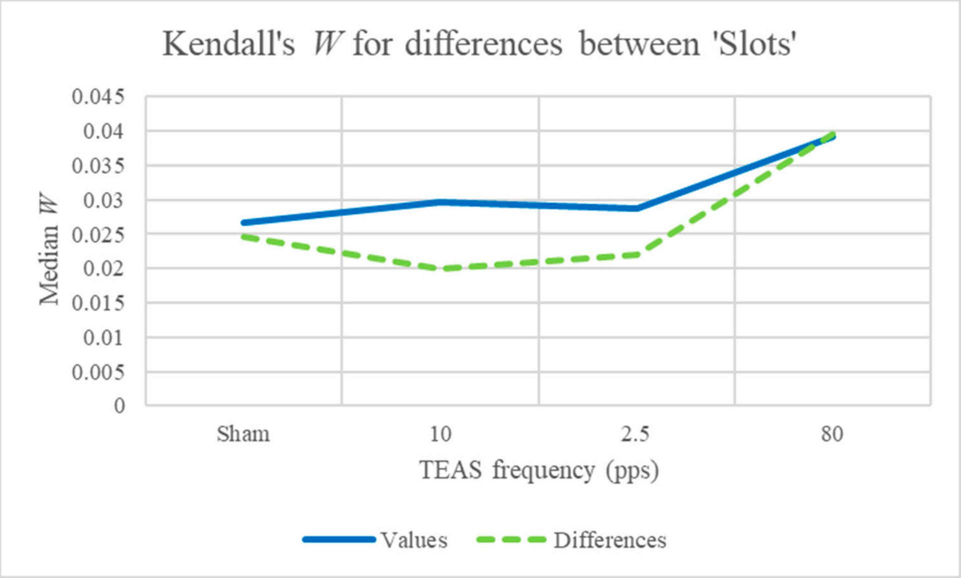

Figure S20. Median effect size (Kendall's *W*) for comparisons between time Slots, for HRV measures at the different stimulation frequencies, using non-normalised and normalised measure Values ('Differences').

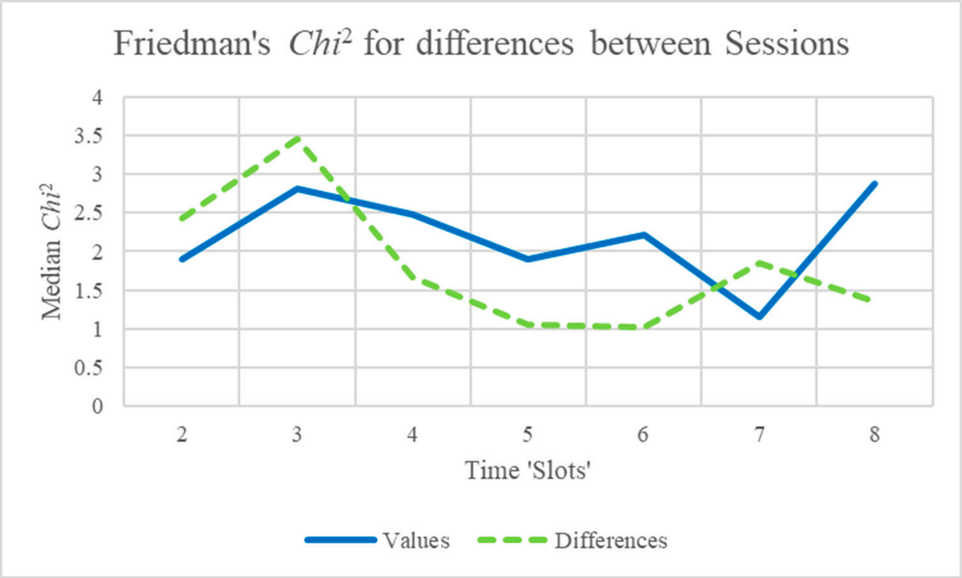

Figure S21. Median values of Friedman's Chi-square for comparisons between Sessions in the different time Slots, for HRV measures at the different stimulation frequencies, using non-normalised and normalised measure Values ('Differences').

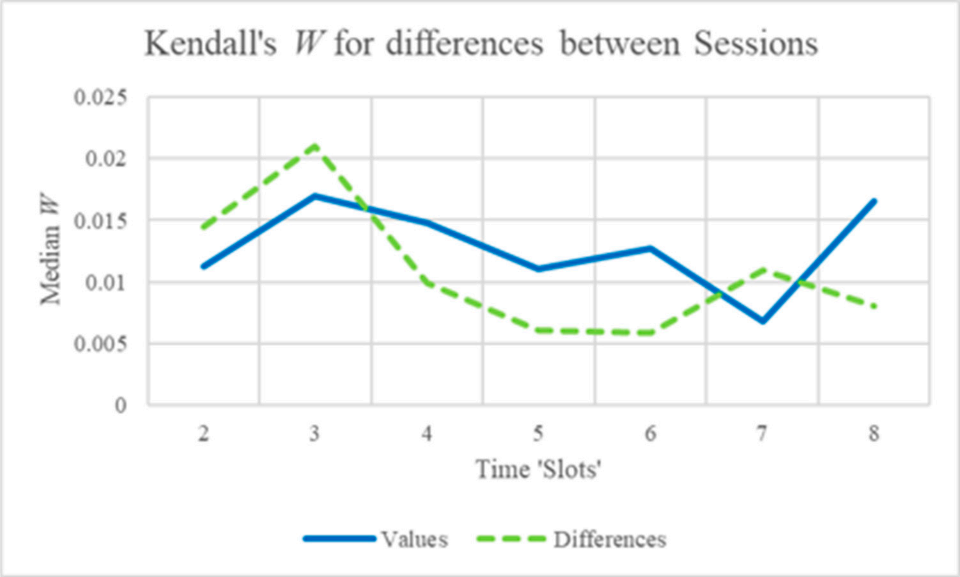

Figure S22. Median effect size (Kendall's *W*) for comparisons between Sessions in the different time Slots, for HRV measures at the different stimulation frequencies, using non-normalised and normalised measure Values ('Differences').

Taking only the Top measures for each comparison, ranking from highest to lowest Differences and Values, **Figures S23-26** juxtapose results for the 'Top 100' BLINKER/CEPS-BLINKER measures and the 'Top 50' HRV measures (only 50, as there were anyway fewer HRV than BLINKER/CEPS-BLINKER measures).

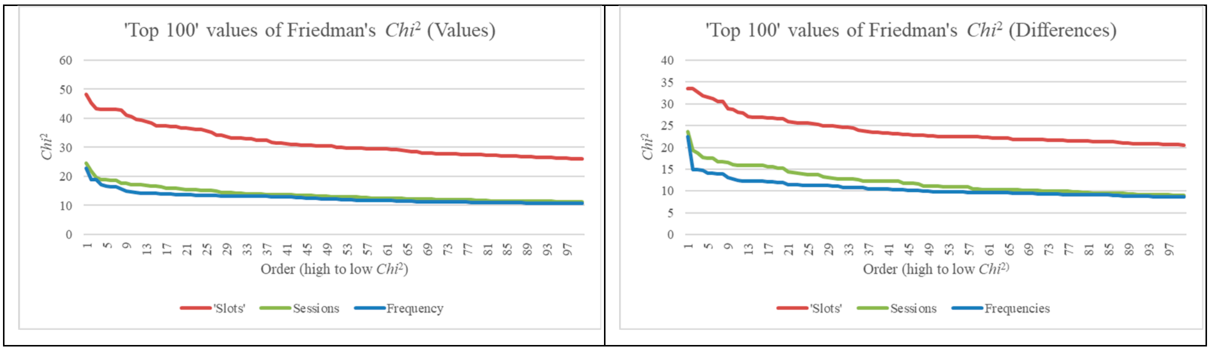

Figure S23. Friedman Chi-square for ‘Top 100’ BLINKER/CEPS-BLINKER measures. **Left**: For Values; **Right**: For Differences.

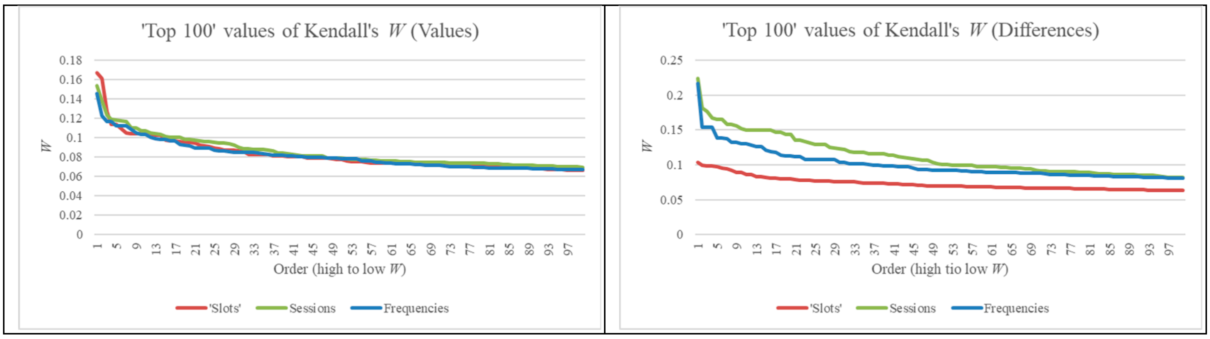

Figure S24. Kendall’s *W* for ‘Top 100’ BLINKER/CEPS-BLINKER measures. **Left**: For Values; **Right**: For Differences.

For the ‘Top 100’ BLINKER/CEPS-BLINKER measures, *Chi*^{2} is greater for Slots than for Stimulation frequencies or Sessions, and greater for Values than for Differences. Results for Kendall’s *W* do not follow the same pattern.

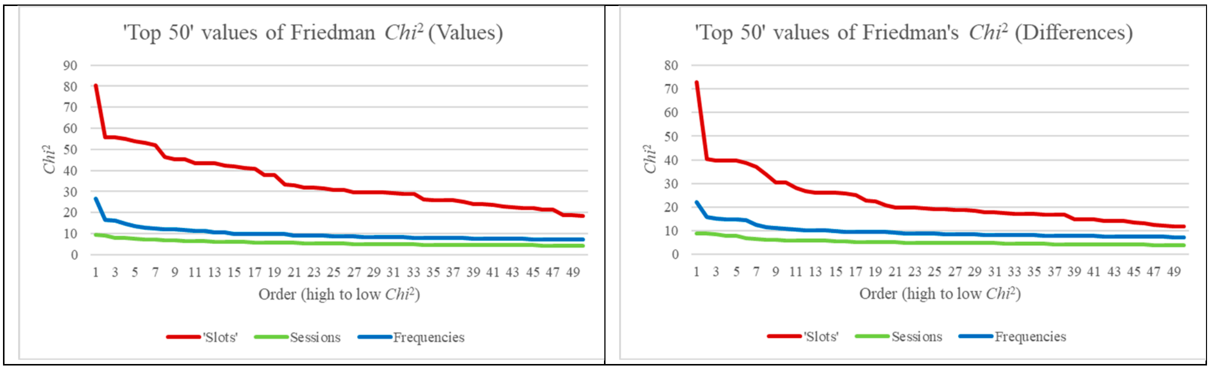

Figure S25. Friedman Chi-square for ‘Top 50’ HRV measures. **Left**: For Values; **Right**: For Differences.

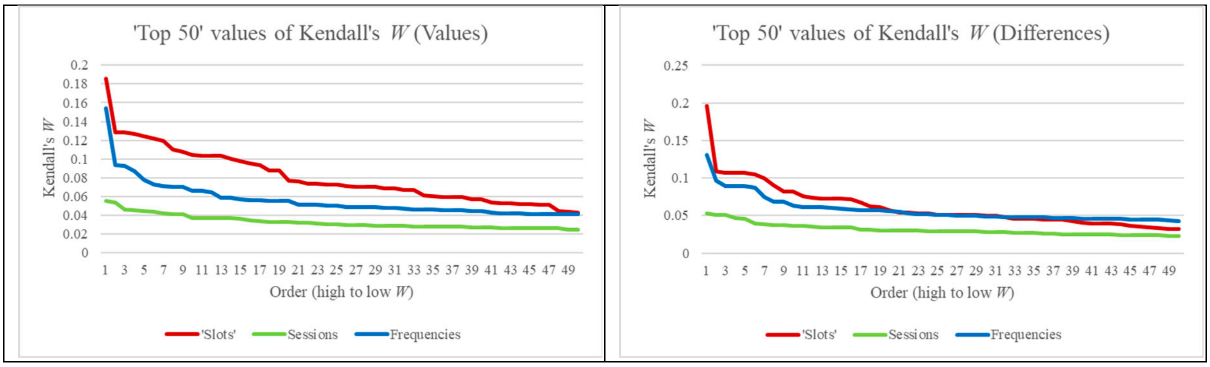

Figure S26. Kendall's *W* for 'Top 50' HRV measures. **Left (A)**: For Values; **Right (B)**: For Differences.

Again, for the 'Top 50' HRV measures, both *Chi*^{2} and *W* are greater for Slots than for Stimulation frequencies or Sessions, and greater for Values than for Differences. Kendall's *W*, on the other hand, is slightly larger for Differences than for Values. **Figures S27** and **S28** summarise these results.

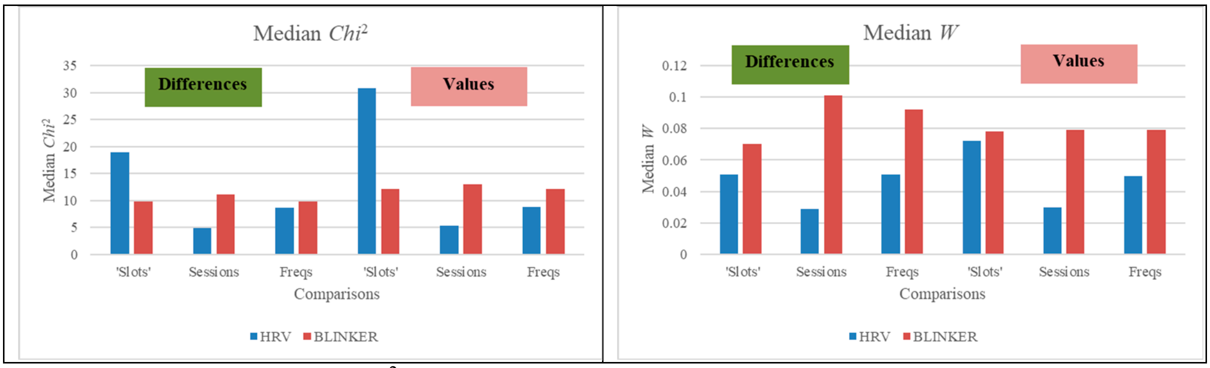

Figures S27 & S28. Median *Chi*^{2} and Kendall's *W* for 'Values' (the values themselves) and 'Differences' (values normalised with respect to baseline). Comparing findings for differences over time within and between sessions, and between stimulation frequencies.

Appendix S3. Exploring the effects of stimulation amplitude

As previously described [20], eight values were taken from the Equinox stimulator amplitude control settings: left and right sensory thresholds (dial reading when participants first became aware of a localised tingling as amplitude was slowly increased), and then left and right tolerances (readings when participants reported stimulation as "strong but comfortable" but not painful). In each session, threshold and tolerance levels were recorded – first on the left hand, then on the right – at the start of the first ten-minute period of stimulation and then half way through, at the start of the second ten-minute period of stimulation (after a short break during which participants completed a numerical rating scale for mood). Averages of the resulting threshold and tolerance levels were used in analysis. The Equinox output was in arbitrary 'dial units' (not in either volts or milliampères). As the device output was set to zero for Sham stimulation, levels were not recorded, even though some participants were aware of it.

For each active (i.e., non-Sham) frequency, amplitude (tolerance) was defined as 'high' or 'low', relative to the group median amplitude for that frequency, and similarly for 'threshold'. **Table S10** shows the group median values for each stimulation frequency.

Table S10. Group median threshold and tolerance values for each stimulation frequency.

Stimulation frequency (pps)	Threshold	Tolerance
2.5 pps	3.00	4.88
10 pps	2.74	3.89
80 pps	2.40	3.18

Note that median values were a function of stimulation frequency, being highest for stimulation at 2.5 pps and lowest for stimulation at 80 pps, with values in between for 10 pps stimulation.

Stimulation amplitude and ocular indices (BLINKER and CEPS-BLINKER measures)

Mann-Whitney tests indicated no significant differences in LRBR (ratio of left to right channels providing best blinks) for data split into two groups with stimulation amplitude threshold or tolerance

high or low relative to their median values. Effect sizes for all frequencies, both during and post-stimulation were < 0.1, the exception being tolerance for 80 pps stimulation, for which effect sizes were > 0,1 (I.e., 'small'), both during and after stimulation (as well as at baseline). Smallest effect size during stimulation (0.024) was for tolerance to 2.5 pps TEAS, largest (0.123) for tolerance to 80 pps. LRBR was consistently > 1.0 for all subgroups.

Median LRBR for the high and low threshold and tolerance subgroups were compared. Despite not being statistically different, a clear pattern emerged: ratios were always greater for the low than for the high threshold or tolerance subgroups, except for 80 pps TEAS tolerance, for which LRBR was greater for the high tolerance subgroup.

In other words, somewhat as found for experimental trigeminal stimulation by Kassem and Evinger [6], blink asymmetries appear to decrease with increasing amplitude (tolerance) of *peripheral* stimulation (TEAS), except for 80 pps TEAS tolerance.

Whereas Mann-Whitney tests indicated no significant differences in LRBR with stimulation amplitude threshold or tolerance, differences were significant for 62 other BLINKER and 965 CEPS-BLINKER measures, as shown in **Table S11** (details available on request).

Table S11. Counts of CEPS-BLINKER (CB) and BLINKER (B) measures that differed significantly (ES > 2) with stimulation threshold and tolerance.

Thresholds	ES > 0.2: *N*	Greater for low T	Greater for high T
Stim 2.5	13	13 CB, 0 B	0
Post 2.5	27	24 CB, 1 B	2 CB. 0 B
Stim 10	27	**8 CB, 18 B**	1 CB, 0 B
Post 10	42	**16 CB, 26 B**	0 CB, 0 B
Stim 80	0	0	0
Post 80	16	16 CB, 0 B	0
**Tolerances**	**ES > 0.2: *N***	**Greater for low T**	**Greater for high T**
Stim 2.5	0	0	0
Post 2.5	0	0	0
Stim 10	29	24 CB, 1 B	3 CB, 1 B
Post 10	23	18 CB, 3 B	1 CB, 1 B
Stim 80	0	0	0
Post 80	0	0	0

Differences with ES > 0.2 occurred more frequently for thresholds than for tolerances, and particularly during and following stimulation at 10 pps. As for LRBR, CEPS-BLINKER and BLINKER measures were in general greater for low rather than high tolerance and threshold. During and after low threshold 10 pps stimulation, there were more increases in BLINKER than in CEPS-BLINKER measures. The BLINKER measures for which this was the case were all variants of duration, apart from one (nAVRZ_goodMedian). The only BLINKER measure that was greater for higher tolerance stimulation was blinksPerMin_goodMean (both during and following 10 pps stimulation).

Appendix S4. Autonomic correlates with the NRS and other data types

HRV measures and NRS scores

Using the method proposed by Vasei et al. [29], NRS mood ratings were considered as either 'positive' (Comfortable, Relaxed Lively) or 'negative' (Anxious, Fatigued, Gloomy), and summed accordingly (omitting Overall Good Mood and Confused). Negative Spearman correlations with p <

0.01 between percentage changes in HRV indices over the course of sessions and *positive* mood at baseline were found for LFlog, LFabs and SD2, suggesting that **a more positive mood at baseline might be associated with a decrease in markers of SNS HRV modulation with stimulation**. When data were split by stimulation frequency, positive correlations with $p < 0.01$ were found between *negative* mood at baseline and subsequent changes in SD2/SD1, relative LF power, LFnu, LF/HF and DFA α1 (all SNS-like indices), but only for 10 pps stimulation, with an increase in the Kubios HRV 'PNS' measure positively correlated with prior negative mood for 80 pps stimulation.

Correlations between the HRV indices and other data type measures

Table S12. Numbers of correlations between the HRV indices and data type measures analysed in this study with absolute values of Spearman's *rho* > 0.3. Numbers are also shown as percentages of all the measures analysed for each data type. In addition, the 2.5th and 97.5th percentiles of the values of Spearman's *rho* are included, in bold type where their absolute values are > 0.3.

	**N *rho* < -0.3**	**% of All**	**2.5%**	**N *rho* > 0.3**	**% of All**	**97.5%**	**All**
MdnPwr Lin	500	**2.72**	**-0.304**	74	0.40	0.219	18360
1 Hz bins	186	1.69	-0.278	70	0.64	0.219	11016
HjorthA	2	0.06	-0.209	11	0.34	0.198	3240
HjorthC	16	0.49	-0.221	264	**8.15**	**0.352**	3240
HjorthM	326	**10.06**	**-0.378**	23	0.71	0.226	3240
Wackermann	42	**3.89**	**-0.316**	6	0.56	0.198	1080
SpCen	268	**13.87**	**-0.369**	22	1.14	0.264	1932
ASYMM	58	0.30	-0.201	122	0.62	0.240	19584
ASYMM Ratio	77	0.34	-0.204	155	0.68	0.246	22848
Reg Ratios	19	0.26	-0.214	84	1.14	0.234	7344
BLINKER	50	0.18	-0.209	55	0.20	0.210	27336
CEPS-BLINKER	845	0.53	-0.239	892	0.56	0.237	160343
Cordance	73	0.66	-0.239	62	0.56	0.236	11016
TEMPSlope	0	0.00	-0.173	2	0.49	0.250	408
**Medians**	65.5	0.51	-0.230	66	0.59	0.235	9180

Correlations between the percentage counts and percentiles are strong for the negative values of *rho* in this Table (Spearman's *rho* =0.941), as found for percentages and counts when 'top slicing' (see main paper). However, for the positive values in the Table above, these correlations were only significant using Pearson's *R* (0.895, $p < 0.001$), not Spearman's *rho*.

Table S13 shows the HRV measures for which |*rho*| > 0.3 in Table 10.

Table S13. The HRV measures for which |*rho*| > 0.3 in Table 10.

	***rho* < -0.3**	***N***	***rho* > 0.3**	***N***
MdnPwr Lin	D2<br>HFabs<br>HFlog<br>LF.Hz<br>LFabs<br>LFlog	12<br>29<br>29<br>15<br>15<br>15	DFA α2<br>ApEn<br>D2<br>HF.Hz<br>SampEn<br>SI	2<br>20<br>1<br>4<br>21<br>24

	NNxx<br>pNNxx<br>RMSSD/SD1<br>SD2<br>SD2/SD1<br>SDHR<br>SDNN<br>TI<br>TINN<br>TotPwr	46<br>39<br>37<br>24<br>1<br>61<br>1<br>37<br>35<br>37	SNS	2
1 Hz bins	D2<br>HFabs<br>HFlog<br>LF.Hz<br>LFabs<br>LFlog<br>NNxx<br>pNNxx<br>PNS<br>RMSSD/SD1<br>RR<br>SD2<br>SD2/SD1<br>SDHR<br>SDNN<br>TI<br>TINN<br>TotPwr	3<br>7<br>7<br>6<br>8<br>8<br>12<br>11<br>1<br>9<br>1<br>10<br>1<br>25<br>16<br>18<br>15<br>19	DFA α2<br>ApEn<br>D2<br>HF.Hz<br>HRmax<br>HRmean<br>HRmin<br>SampEn<br>SDHR<br>SI<br>SNS	8<br>23<br>1<br>6<br>2<br>1<br>4<br>9<br>1<br>13<br>2
HjorthA	SD2/SD1<br>DFA α1	1<br>1	DFA α2<br><br>ApEn<br>SampEn	1<br>7<br>3
HjorthC	ApEn<br>RR<br>SI	1<br>1<br>14	D2<br>HFabs<br>HFlog<br>HRmean<br>LFabs<br>LFlog<br>NNxx<br>pNNxx<br>RMSSD/SD1<br>SD2<br>SDHR<br>SDNN<br>TI<br>TINN<br>TotPwr	13<br>5<br>5<br>1<br>12<br>12<br>19<br>15<br>16<br>19<br>37<br>26<br>24<br>23<br>21
HjorthM	D2<br>HFabs<br>HFlog<br>LFabs<br>LFlog<br>NNxx<br>pNNxx	19<br>14<br>14<br>15<br>15<br>29<br>24	SD2/SD1<br>SI	2<br>21

	RMSSD/SD1	22		
	SD2	19		
	SDHR	31		
	SDNN	26		
	TI	28		
	TINN	25		
	TotPwr	23		
Wackermann	DFA α2	1	ApEn	1
	D2	2	SampEn	1
	HFabs	1	SI	4
	HFlog	6		
	LFabs	6		
	LFlog	6		
	RMSSD/SD1	1		
	SD2	4		
	SDHR	3		
	SDNN	4		
	TI	4		
	TINN	4		
	TotPwr	4		
SpCen	D2	12	DFA α2	1
	HFabs	7	SI	21
	HFlog	7		
	LFabs	16		
	LFlog	16		
	NNxx	19		
	pNNxx	19		
	RMSSD/SD1	20		
	SD2	20		
	SDHR	20		
	SDNN	25		
	TI	24		
	TINN	21		
	TotPwr	22		
ASYMM	ApEn	2	DFA α1	8
	HF.Hz	1	ApEn	2
	HFabs	3	D2	6
	HFlog	3	HFabs	7
	HFrel	2	HFlog	7
	HRmax	2	HRmax	3
	HRmean	2	HRmean	1
	HRmin	2	HRmin	3
	LF.Hz	4	LF.Hz	1
	NNxx	5	LFabs	7
	pNNxx	5	LFlog	7
	PNS	10	NNxx	7
	RMSSD	5	pNNxx	6
	RR	1	RMSSD/SD1	3
	SampEn	1	RR	2
	SD1	5	SD2	6
	SI	5	SD2/SD1	12
			SDHR	5
			SDNN	6
			SNS	1

			TI<br>TINN_ms<br>TotPwr	7<br>5<br>7
ASYMM Ratio	ApEn<br>D2<br>HF.Hz<br>HFabs<br>Hflog<br>Hfnu<br>HFrel<br>HRmax<br>HRmin<br>LF.Hz<br>LF/HF<br>LFnu<br>LFrel<br>NNxx<br>pNNxx<br>PNS<br>RMSSD/SD1<br>RR<br>SampEn<br>SD2<br>SDHR<br>SDNN<br>SI<br>TI<br>TINN<br>TotPwr	4<br>1<br>1<br>3<br>3<br>2<br>3<br>1<br>1<br>2<br>1<br>1<br>1<br>7<br>7<br>11<br>6<br>2<br>1<br>1<br>1<br>2<br>4<br>2<br>2<br>1	DFA α1<br>DFA α2<br>ApEn<br>D2<br>HF.Hz<br>HFabs<br>HFlog<br>Hfnu<br>HFrel<br>HRmax<br>HRmean<br>HRmin<br>LF.Hz<br>LF/HF<br>LFabs<br>LFlog<br>Lfnu<br>LFrel<br>NNxx<br>pNNxx<br>RMSSD/SD1<br>SD2<br>SD2/SD1<br>SDHR<br>SDNN<br>SI<br>SNS<br>TI<br>TINN_ms<br>TotPwr	6<br>1<br>3<br>6<br>1<br>8<br>8<br>1<br>1<br>6<br>2<br>5<br>1<br>2<br>9<br>9<br>2<br>3<br>6<br>5<br>3<br>8<br>10<br>8<br>7<br>2<br>4<br>11<br>6<br>8
Reg Ratios	D2<br>HF.Hz<br>HFnu<br>HFrel<br>HRmean<br>HRmin<br>LF.Hz<br>NNxx<br>pNNxx<br>RMSSD/SD1<br>RR<br>SampEn<br>SDHR<br>SI	2<br>1<br>1<br>1<br>1<br>1<br>1<br>1<br>1<br>1<br>2<br>1<br>2<br>2	ApEn<br>D2<br>HF.Hz<br>HFabs<br>HFlog<br>HRmax<br>HRmean<br>HRmin<br>LF.Hz<br>LFabs<br>LFlog<br>NNxx<br>pNNxx<br>RMSSD/SD1<br>RR<br>SampEn<br>SDHR<br>SDNN<br>TI<br>TINN<br>TotPwr	1<br>7<br>2<br>8<br>8<br>2<br>2<br>1<br>3<br>3<br>3<br>7<br>6<br>6<br>1<br>1<br>2<br>2<br>2<br>3<br>2

BLINKER	DFA α1	1	DFA α2	6
	ApEn	8	HF.Hz	1
	D2	4	HRmax	2
	HFabs	1	LF.Hz	2
	HFlog	1	LFabs	3
	HRmean	5	LFlog	3
	HRmin	4	NNxx	2
	NNxx	4	pNNxx	2
	pNNxx	4	RMSSD	4
	RMSSD/SD1	3	RR	5
	SD2/SD1	4	SampEn	1
	SI	4	SD2	3
	SNS	2	SDHR	7
	TI	2	SDNN	4
			TI	2
			TINN_ms	2
			TotPwr	2
CEPS-BLINKER	DFA α1	12	DFA α1	3
	DFA α2	45	DFA α2	89
	ApEn	12	ApEn	4
	D2	79	D2	59
	HF.Hz	14	HF.Hz	17
	HFabs	70	HFabs	59
	HFlog	70	HFlog	59
	HFnu	4	HFnu	7
	HFrel	4	HFrel	7
	HRmax	7	HRmax	16
	HRmean	11	HRmean	9
	HRmin	16	HRmin	4
	LF.Hz	6	LF.Hz	4
	LF/HF	7	LF/HF	4
	LFabs	12	LFabs	6
	LFlog	12	LFlog	6
	LFnu	7	LFnu	4
	LFrel	8	LFrel	3
	NNxx	60	NNxx	65
	pNNxx	60	pNNxx	57
	PNS	15	PNS	21
	RMSSD/SD1	67	RMSSD/SD1	84
	RR	9	RR	11
	SampEn	4	SampEn	10
	SD2	22	SD2	18
	SD2/SD1	14	SD2/SD1	9
	SDHR	17	SDHR	34
	SDNN	18	SDNN	25
	SI	19	SI	14
	SNS	26	SNS	15
	TI	23	TI	39
	TINN	18	TINN	19
	TotPwr	18	TotPwr	22
Cordance	DFA α1	9	DFA α1	4
	DFA α2	1	DFA α2	3
	D2	2	ApEn	2
	HFabs	4	D2	2

	HFlog<br>HRmax<br>HRmean<br>HRmin<br>LF.Hz<br>LF/HF<br>LFabs<br>LFlog<br>LFnu<br>LFrel<br>NNxx<br>PNS<br>RMSSD<br>RR<br>SD2<br>SD2/SD1<br>SDHR<br>SDNN<br>SNS<br>TI<br>TINN<br>TotPwr	4<br>3<br>2<br>1<br>1<br>6<br>1<br>1<br>5<br>6<br>1<br>3<br>1<br>1<br>1<br>5<br>7<br>1<br>2<br>1<br>2<br>1	HFabs<br>HFlog<br>HFnu<br>HFrel<br>HRmax<br>HRmean<br>HRmin<br>LF.Hz<br>NNxx<br>pNNxx<br>PNS<br>RMSSD<br>RR<br>SampEn<br>SD2/SD1<br>SI<br>TotPwr	6<br>6<br>6<br>7<br>1<br>1<br>1<br>3<br>2<br>3<br>5<br>2<br>2<br>1<br>1<br>1<br>1
TEMPSlope	n/a		LFlog<br>LFabs	1<br>1

The most commonly occurring PNS-like HRV indices in Table S13 are NNxx (292 occurrences), RMSSD (290), pNNxx (264), HFlog (237), HFabs (232) and D2 (231) In contrast, the single most commonly occurring SNS index is SI (148 occurrences). SDHR is the only ‘ambivalent’ index occurring more than 200 times.

For the different data types, the four correlations with highest positive and negative values of *rho* are shown in **Table S14**.

Table S14. Top and bottom two correlations between data type measures and HRV indices.

	**Data type**	***rho* < -0.3**	**HRV**	**Data type**	***rho* > 0.3**	**HRV**
MdnPwr<br>Lin	Beta<br>Beta1	C4<br>C4	SDHR<br>SDHR	O20<br>O20	Fz<br>Fz	ApEn<br>ApEn
1 Hz bins	2.5_I_PS_mT<br>20.0_I_PS_mT	F4<br>C4	LF.Hz<br>TotPwr	2.5_I_PS_mT<br>2.5_I_PS_mT	Pz<br>P3	HF.Hz<br>ApEn
HjorthA	HjorthA<br>HjorthA	Fz<br>Fz	SD2/SD1<br>DFA α1	HjorthA<br>HjorthA	C4<br>P3	Apen<br>ApEn
HjorthC	HjorthC<br>HjorthC	F3<br>C4	SI<br>SI	HjorthC<br>HjorthC	C4<br>C3	SDHR<br>SDHR
HjorthM	HjorthM<br>HjorthM	Cz<br>Pz	SDHR<br>SDNN	HjorthM<br>HjorthM	Pz<br>P4	SI<br>SI
Wackerman<br>n	Median Phi		TI<br>SDNN	Median Sigma<br>Median Phi		ApEn<br>SI
SpCen	SpCen	Pz<br>Pz	SDNN<br>TotPwr	SpCen	Pz<br>Pz	SI<br>SI
ASYMM	Beta1<br>Beta	Pz_Fz<br>Pz_Fz	PNS<br>PNS	Beta1<br>Beta	Pz_Fz<br>Pz_Fz	SD2/SD1<br>SD2/SD1

ASYMM Ratio	Beta1_r<br>Beta_r	Pz_Fz<br>Pz_Fz	PNS<br>PNS	Beta1_r<br>Beta_r	Pz_Fz<br>Pz_Fz	SD2/SD1<br>SD2/SD1
Reg Ratios	Alpha_a<br>Beta1_r	P_A<br>C_O	SI<br>NNxx	Alpha_r<br>Alpha_a	C_O<br>P_A	D2<br>LFlog
BLINKER	dB_goodMedian<br>nAVRZ_goodMed ian		ApEn<br>ApEn	dHB_goodMa d<br>pAVRZ_std		SDHR<br>SDHR
CEPS-BLINKER	leftR2<br>leftR2	Kurtosis<br>Kurtosis	NNxx<br>RMSSD/ SD1	y_int<br>leftSlope	CCM<br>Skewness	HF.Hz<br>RMSSD/S D1
Cordance	SQzTInf_mT Beta<br>SQzTInf_mT Beta	C4<br>C4	TotPwr<br>HFabs	SQzTInf_mT Alpha	Fz<br>Fz	pNNxx<br>NNxx
TEMPSlope	n/a			TEMPSlope		LFlog<br>LFabs

Of the HRV indices in the above Table, 14 can be categorised as SNS-like, 12 PNS-like, 13 ‘ambivalent’ and 10 as ‘other’.

Results for the different data types were examined using Mann-Whitney tests (with p-values < 0.001) to determine their dependence on Sex (Female/Male) or binarised (‘high’/’low’) factors such as Age, PSS ‘coping’ and ‘distress’ scores, or the ‘positive’ and ‘negative’ NRS mood ratings proposed by Vasei et al. [29]. Our expectation was that those data type measures having the strongest correlations with the HRV PNS-like and SNS-like indices would also show larger effect sizes for differences between ‘high’ and ’low’ ‘coping’ or ‘distress’, and for differences between ‘positive’ and ‘negative’ NRS scores, with lower effect sizes for sex or age.

With data split into 12 subgroups (4 stimulation frequencies × 3 phases – Pre [Slot 1], Stim [Slots 2-5] and Post [Slots 6-8]) to at least partially mitigate against non-independence of data when calculating the correlations, outcomes were not as expected.

For PSS-D, four measures occurred with p <0.001 (three time during Stim and once Post-stim, not at all at baseline), with effect size between 0.2 and 0.3. For PSS-C, only one measure occurred more than once (three times during and twice following stimulation), with effect size between 0.3 and 0.4. No measures occurred more than once for the binarised NRS scores (effect size between 0.2 and 0.3).

In contrast, for sex, the Pz Spectral centroid occurred 7 times during and after stimulation (3 times with effect size between 0.3 and 0.4), and the Beta1 central/outer asymmetry ratio four times (but only with effect size between 0.2 and 0.3). For Age, 7 measures occurred 10 times or more, with stronger effects sizes, sometimes even > 0.5 *at baseline*. At C4, Beta and Beta1 power, as well as cordance (SQzTInf_mT), often resulted in ES > 0.4. The Hjorth complexity and mobility parameters for the central electrodes (C3, Cz, C4) and at Pz also differed with age. **Table S15** shows some results.

Table S15. Counts of differences in data type measures with age, ES > 0.4.

**Age**	**Pre**	**Stim**	**Post**	**Sums**
Sham	3	3	6	12
2.5 pps	3	8	9	20
10 pps	4	4	9	17
80 pps	5	7	8	20
Sums	15	22	32	69

Age has less effect on the various data type measures at Baseline, or for Sham stimulation, than during or after stimulation. **Figure 29** illustrates overall differences with age for two of the Hjorth parameters.

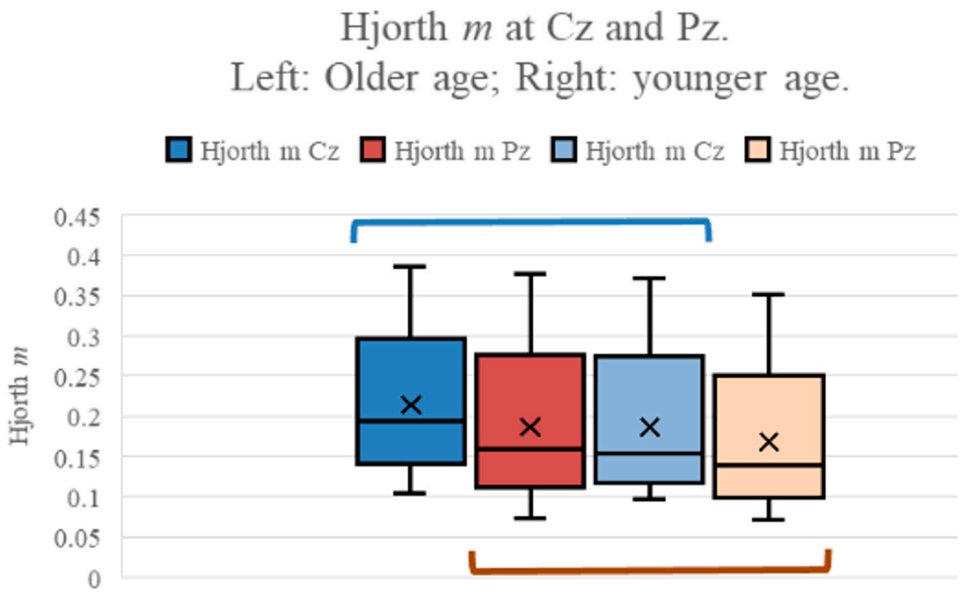

Figure 29. Differences with age in HjorthM at electrodes Cz and Pz (data unsplit).

Abbreviations used in the Supplementary Material but not the main paper

Table S16. Other abbreviations used in this Supplementary Material but not in the main paper.

**ECG/PPG**	**Measure or term**	**Abbreviation**
	Percentage change (trend) in 3- or 6-second segments of ECG or PPG data (as in 'D3_trend_pc_BVP1', 'D6_trend_pc_ECG1'); '1' = Right hand; '2' = 'Left hand'	trend_pc
**HRV**	**Measure or term**	**Abbreviation**
	Peak frequency in HRV low frequency band	LF.Hz
**EEG**	**Measure or term**	**Abbreviation**
	Anterior, central, left (as in 'xPWR_L8', x coordinate of L power centroid, or 'xFREQ_A7', x coordinate of L frequency centroid)	_A, _C, _L (suffix)
	Asymmetry (Asymmetry ratio), as in 'Mdn Pr A' ('Mdn Pr AR')	A (AR)
	Absolute, Relative (EEG power, as in 'Alpha_a', 'Alpha_r'))	_a, _r (suffix)
	Absolute spectral power	ASP (but not RSP)
	Hjorth Activity in an EEG channel (as in 'HjorthA_Fz_1') [meaning of '1' mislaid]	HjorthA_[channel]_1
	Hjorth Complexity in an EEG channel (as in 'HjorthC_C4')	HjorthC_[channel]
	Central-Outer (or Peripheral-Central), Posterior-Anterior (as in 'Theta_C_O_r' or 'Periph_Cent_O5_r', 'Theta_sham_P_A_a ')	C_O (or P_C, Periph-Cent), P_A (or Post_Ant)

	Spectral centroid (as in 'SpCen_P4_0', in channel P4 for sham stimulation)	SpCen
	Square-root normalised or normalised and Z-transformed (as in 'SQNoAM_mT_Theta_F3', 'SQzTInf_mT Alpha')	SQzNo, SQzT
	Wackermann Sigma at epoch number, AMICA	Sigma_[number 1 to 75]_A
	Channel pair examples	Pz_Fz, P4_O1
	Thea Radüntz (as in 'Phi_55_I_TR', Wackermann Phi at epoch number 55, ICA using InfoMax, EEG data processed using TR's semi-automated machine learning method)	TR
	Log normalised (as in 'LNNoAM_mT_Alpha_C4')	LNNo
	Log normalised and Z-transformed (as in 'LNzTAM_mT_Theta_P8')	LNzT
	AMICA	AM
	Complex Morlet wavelet, alternative to Fourier tranform for time-frequency analysis (as in 'LNNoInf_CMW_[band]_[channel]')	CMW
**Temperature**	**Measure or term**	**Abbreviation**
	Temperature slope normalised (Differences) and values (not normalised)	TempSlope or TempSlope V
**CEPS**	**Measure or term**	**Abbreviation**
	Shimmer (3- or 5-point amplitude perturbation quotient)	apq3, apq5
	Jitter (Relative Average Perturbation)	RAP
	Permutation_Jensen-Shannon_Complexity	PJSC
	Phase entropy	PhEn
**Other**	**Measure or term**	**Abbreviation**
	Perceived stress scale (Coping, Distress)	PSS-C, PSS-D
	Session, 'Slot', Stimulation frequency	Se, Sl, St
**Statistics**	**Measure or term**	**Abbreviation**
	Z-score	Z
	Standard deviation	std
